# Galunisertib plus gemcitabine vs. gemcitabine for first-line treatment of patients with unresectable pancreatic cancer

**DOI:** 10.1038/s41416-018-0246-z

**Published:** 2018-10-15

**Authors:** Davide Melisi, Rocio Garcia-Carbonero, Teresa Macarulla, Denis Pezet, Gael Deplanque, Martin Fuchs, Jorg Trojan, Helmut Oettle, Mark Kozloff, Ann Cleverly, Claire Smith, Shawn T. Estrem, Ivelina Gueorguieva, Michael M. F. Lahn, Al Blunt, Karim A. Benhadji, Josep Tabernero

**Affiliations:** 1 University of Verona, Piazzale Ludovico Antonio Scuro, 10, 37134 Verona, Italy; 2 Oncology Department, Hospital Universitario 12 de Octubre, Instituto de Investigación Sanitaria Hospital 12 de Octubre (imas12), UCM, CNIO, CIBERONC, Madrid, Spain; 3 Vall d’Hebron University Hospital Institute of Oncology (VHIO), CIBERONC, C/ Natzaret, 115-117, 08035 Barcelona, Spain; 4 Centre Hospitalier Universitaire, 1 Place Lucie Aubrac, 63003 Clermont-Ferrand, France; 5 Hôpital Riviera-Chablais, Avenue de la Prairie 3, 1800 Vevey, Switzerland; 6 Klinikum Bogenhausen, Städtisches Klinikum München GmbH, Englschalkinger Road 77, 81925 Munich, Germany; 7 Goethe University Medical Center, Theodor-Stern-Kai 7, 60590 Frankfurt, Germany; 8 Onkologische und Hämatologische Schwerpunktpraxis, Friedrichshafen, Germany; 9 Ingalls Memorial Hospital, 71W. 156th St., Harvey, IL 60426 USA; 10 Eli Lilly and Company, Erl Wood Manor, Windlesham, Surrey, GU20 6PH UK; 11 formerly of Eli Lilly and Company, Indianapolis, IN 46285 USA; 12 Eli Lilly and Company, Lilly Corporate Center, Indianapolis, IN 46285 USA; 13 6820 Wisconsin Ave., #8008, Bethesda, MD 20815 USA; 14 Advaxis, Inc., 305 College Road East, Princeton, NJ 08540 USA; 15 Eli Lilly and Company, 440 Route 22 East, Bridgewater, NJ 08807 USA; 16 Vall d’Hebron University Hospital and Institute of Oncology (VHIO), CIBERONC, Universitat Autònoma de Barcelona, P. Vall d’Hebron 119-129, 08035 Barcelona, Spain

**Keywords:** Cancer immunotherapy, Prognostic markers, Predictive markers

## Abstract

**Background:**

Galunisertib is the first-in-class, first-in-human, oral small-molecule type I transforming growth factor-beta receptor (ALK5) serine/threonine kinase inhibitor to enter clinical development. The effect of galunisertib vs. placebo in patients with unresectable pancreatic cancer was determined.

**Methods:**

This was a two-part, multinational study: phase 1b was a non-randomised, open-label, multicentre, and dose-escalation study; phase 2 was a randomised, placebo- and Bayesian-augmented controlled, double-blind study in patients with locally advanced or metastatic pancreatic adenocarcinoma considered candidates for first-line chemotherapy with gemcitabine. Patients were randomised 2:1 to galunisertib–gemcitabine (*N* = 104) or placebo-gemcitabine (*N* = 52). Gemcitabine dose was 1000 mg/m^2^ QW. Primary endpoints for phases 1b and 2, respectively, were phase 2 dose and overall survival. Secondary objectives included tolerability and biomarkers.

**Results:**

Dose-escalation suggested a 300-mg/day dose. Primary objective was met: median survival times were 8.9 and 7.1 months for galunisertib and placebo, respectively (hazard ratio [HR] = 0.79 [95% credible interval: 0.59–1.09] and posterior probability HR < 1 = 0.93). Lower baseline biomarkers macrophage inflammatory protein-1-alpha and interferon-gamma-induced protein 10 were associated with galunisertib benefit.

**Conclusions:**

Galunisertib–gemcitabine combination improved overall survival vs. gemcitabine in patients with unresectable pancreatic cancer, with minimal added toxicity. Future exploration of galunisertib in pancreatic cancer is ongoing in combination with durvalumab.

## Background

Pancreatic cancer remains one of the most lethal and poorly understood human malignancies.^1^ It has the lowest 5-year relative survival rate among solid tumours at 8%^2^ and is projected to become the second leading cause of cancer-related death by 2030 in Western countries.^3^ Poor prognosis in pancreatic cancer is attributed to its early metastatic behaviour, aggressive clinical course, and limited efficacy of chemotherapeutic treatments.^4,5^

The transforming growth factor-beta (TGF-β) signalling pathway has one of the most complex and controversial roles in cancer. TGF-β maintains homoeostasis in normal tissue; however, cancer cells have the capacity to circumvent this suppressive influence. Thus, pathological forms of TGF-β signalling promote tumour growth, epithelial-to-mesenchymal transition, extracellular matrix remodelling, stemness and evasion of immune surveillance.^6^ Recent whole-genome or exome sequencing confirmed TGF-β as the most recurrently mutated signal transduction pathway in pancreatic cancer.^7^

Inhibitors of TGF-β signalling have been explored in pre-clinical models and showed enhanced anti-tumour activity in combination with gemcitabine.^8^ Galunisertib is the first orally bioavailable small-molecule inhibitor of the type I TGF-β receptor (ALK5) serine/threonine kinase to enter clinical development.^9^

The present study was designed to determine an appropriate dose of galunisertib for combination with gemcitabine and to evaluate the combination for treatment of pancreatic cancer. Safety, pharmacokinetics, anti-tumour activity and biomarkers related to pancreatic cancer and TGF-β signalling were also evaluated.

## Methods

### Study design and participants

This was a multinational, two-part study of oral galunisertib in combination with gemcitabine. The first part (phase 1b) was a non-randomised, open-label, multicentre, dose-escalation phase in patients with solid malignancies who had not responded to anti-cancer therapies and who were eligible for gemcitabine therapy. The second part (phase 2) was a randomised, placebo- and Bayesian-augmented controlled, double-blind study of galunisertib in combination with gemcitabine vs. gemcitabine plus placebo in patients with locally advanced or metastatic pancreatic adenocarcinoma at first presentation or after recurrence following radical surgery, and who were considered candidates for first-line chemotherapy with gemcitabine (Supplementary Figure S1). Previous adjuvant or neoadjuvant gemcitabine was allowed. Additional details in the Supplementary Materials.

### Randomisation and masking

In phase 2, patients were randomly assigned 2:1 in the galunisertib group vs. the placebo group, using a dynamic randomisation procedure^10^ to minimise imbalance between treatment groups using the known prognostic factors of Eastern Cooperative Oncology Group (ECOG) performance (0 vs. 1 vs. 2), disease stage (stages II–III vs. IV), and previous gemcitabine treatment (adjuvant/neoadjuvant vs. no treatment), as well as investigational site.

### Procedures

Patients were treated orally twice daily (morning and evening) with galunisertib at dose levels of 80, 160 or 300 mg/day during phase 1b and 300 mg/day or matching placebo during phase 2 for 14 days followed by 14 days off in a 28-day cycle. All patients (phase 1b and 2) received gemcitabine via intravenous infusion at a dose of 1000 mg/m^2^ over 30 min (~ 60 min maximum) once weekly for 7 weeks, followed by a week of rest from treatment. Given the selected centres, this regimen of gemcitabine was the most consensual at the time of study initiation. In order to characterise the galunisertib pharmacokinetic profile, the initial dose of gemcitabine was administered 7 days (±3 days) after the first dose of galunisertib or placebo was started. Subsequent gemcitabine cycles consisted of infusions once weekly for 3 of every 4 weeks. Additional details in the Supplementary Materials.

### Outcomes

In phase 1b, the primary objective was to determine a safe/tolerable phase 2 dose of galunisertib in combination with gemcitabine using a 3 + 3 dose-escalation design. The phase 2 dose was expected to achieve a pre-defined plasma galunisertib exposure that was below levels associated with pre-clinical cardiovascular toxicity.^11^

In phase 2, the primary objective was to compare overall survival in the galunisertib and placebo groups using a Bayesian analysis. Key secondary objectives included evaluation of the pharmacokinetic profile and tolerability of galunisertib, comparison of the treatment groups with respect to progression-free survival and overall response rate as assessed by the investigators and central reviewers, evaluation of biomarker changes in tumour tissue and circulating blood, and assessment of patient-reported pain using the Brief Pain Inventory-short form (BPI-sf) and investigator-rated analgesic level.

### Statistical analysis

Approximately 150 patients were to be randomly assigned and the final analysis/evaluation of overall survival was to be performed after approximately 135 events (deaths) had been recorded or 18 months after the last patient was enroled, whichever was sooner. The pre-specified Bayesian-augmented design consisted of borrowing data from trials in which patients with similar characteristics received the control treatment to optimise treatment comparison of interest and minimise the number of patients enroled in the concurrent control group. Using patient-level control data from two previous randomised gemcitabine trials^12,13^ and assuming a hazard ratio [HR] of 0.7 for survival, the study had 90% power to identify if the probability of HR < 1 was >0.85. The type 1 error rate was 0.16.

Phase 2 efficacy analyses were conducted on the intention-to-treat population, unless otherwise specified. This population included all patients randomly assigned to study treatment. The primary method of analysis for comparing overall survival between the treatment groups used a Bayesian exponential-likelihood model with a hierarchical random-effects distribution on treatment effects. Additional details in the Supplementary Materials.

Sensitivity analyses included an additional Bayesian analysis with minimal borrowing of historical data for the control. In addition, to assess whether unexpected bias was introduced in this trial design, frequentist analyses of overall survival and progression-free survival using only the data from this study were conducted. Efficacy analyses of overall survival and progression-free survival used Kaplan–Meier estimates and the log-rank test stratified by randomisation factors of Eastern Cooperative Oncology Group (ECOG) status, previous gemcitabine treatment, and disease stage (as assessed by investigator and confirmed by central re-read) (Supplementary Table S1). Cox proportional hazards models also adjusted for these randomisation factors, and stepwise analysis adjusted for multivariate factors including ECOG, previous gemcitabine treatment, baseline liver metastasis, sex, post-discontinuation systemic therapy and CA19-9. Overall response rates (complete response + partial response), clinical benefit rates (complete response + partial response + stable disease) and the exact 95% confidence interval (CI) were estimated for each group. The comparison between groups was done using the Fisher’s exact test. All Bayesian analyses were carried out using the statistical software FACTS v2.4 (Berry Consultants LLC, Austin, TX) and all other analyses were carried out using SAS/Version 9.4.

Details on pharmacokinetics and exploratory analyses on circulating biomarkers are available in the Supplementary Materials.

## Results

This phase 1b/2 study was conducted between June 2011 and December 2016 at 24 centres in six countries. Of the 199 patients who entered screening for phase 2, 43 were screen-failures and 156 were randomised to study treatment, yielding 104 and 52 in the galunisertib and placebo intention-to-treat populations, respectively (Supplementary Figure S1). One patient assigned to galunisertib withdrew prior to receiving treatment, and 103 patients in the galunisertib group and 52 in the placebo group received at least one dose of study treatment (Supplementary Figure S2).

In phase 2, 87 (56%) patients had an ECOG status of 1 and 151 (97%) patients had stage III/IV disease at study entry (Supplementary Table S2). Within high-enroling centres, the dynamic randomisation successfully ensured balance between the treatments (data not shown). A median of 2.0 (range: 0–29) and 1.0 (range: 0–19) cycles were completed per patient for galunisertib and gemcitabine, respectively. Dose omissions occurred for galunisertib in 74 patients and for gemcitabine in 96 patients; dose reductions occurred for galunisertib in 14 patients and gemcitabine in 68 patients; dose discontinuations occurred for galunisertib for 34 patients and dose delays occurred for gemcitabine in 30 patients. A total of 84 patients died in the galunisertib group and 48 patients died in the placebo group.

**Fig. 1:**
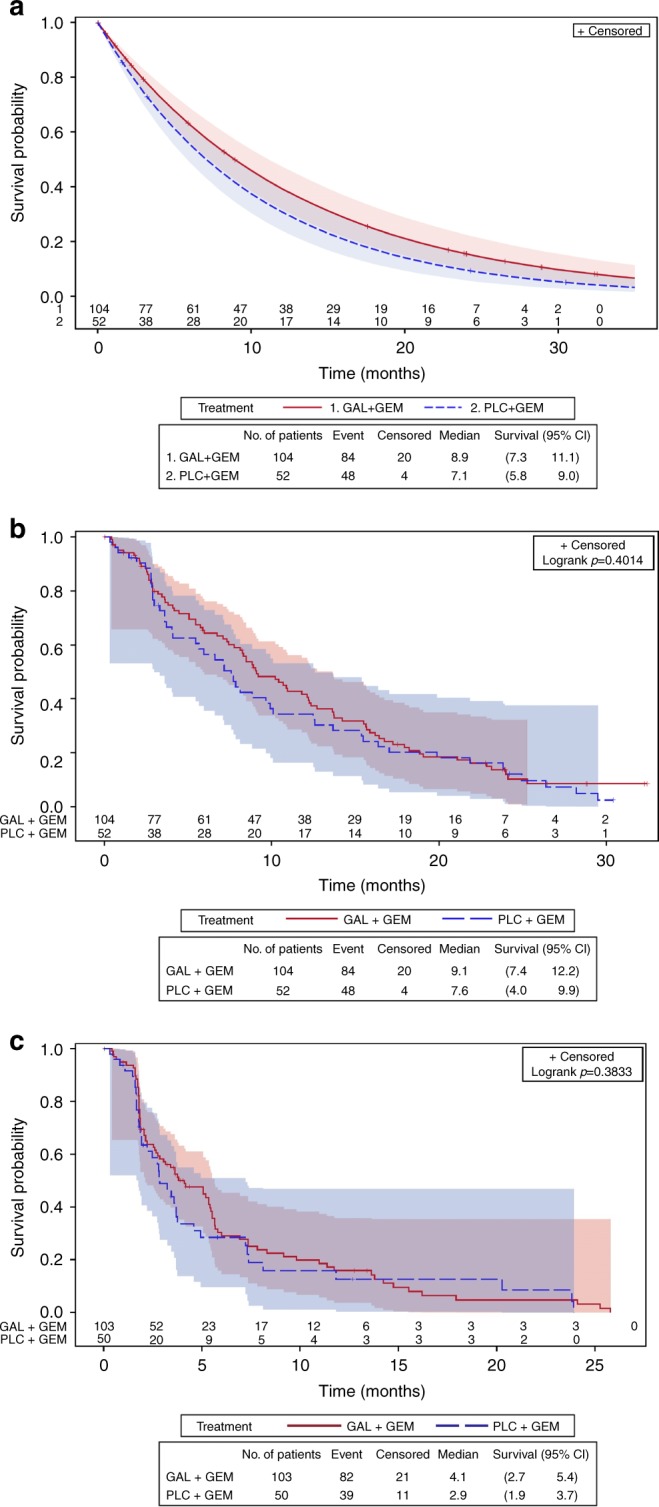
Kaplan–Meier estimates of survival by Bayesian vs. frequentist analysis, with 95% Hall–Wellner confidence bands and numbers at risk. overall survival by **a** Bayesian analysis, **b** frequentist analysis and **c** progression-free survival by frequentist analysis. Data for one patient was missing from the progression-free survival analysis. CI=confidence interval; GAL=galunisertib; GEM=gemcitabine; No.=number; PLC=placebo

From the primary Bayesian analysis, the median overall survival was 7.1 months (95% credible interval [CrI]: 5.8–9.0) for the placebo group and 8.9 months (95% CrI: 7.3–11.1) for the galunisertib group (Fig. 1a). The posterior median HR for overall survival was 0.79 (95% CrI: 0.59–1.09) (Table 1). The posterior probability that the HR is <1 was 0.93, thus the primary objective was met.

**Table 1:** Overall survival analysis for phase 2

Statistical analysis method	Median OS (95% CrI or CI)^a^		Hazard ratio (95% CrI or CI)^a^	Posterior probability *P* (HR < 1)
	Placebo Group^b^ (*N* = 52)	Galunisertib Group^c^ (*N* = 104)	Galunisertib vs. Placebo	
*Bayesian analysis*				
Primary analysis **S**ee Fig. 2a: Bayesian model with strong borrowing of historical data **(**~ 37 events**)**	7.1 (5.8–9.0)	8.9 (7.3–11.1)	0.79 (0.59–1.09)	0.93
*Sensitivity analysis*				
Secondary analysis Bayesian model with weak borrowing of historical data (~32 events)	7.4 (5.7–10.0)	8.9 (7.2–11.1)	0.83 (0.59–1.19)	0.84
Frequentist analysis **S**ee Fig. 2b: Kaplan–Meier method with log-rank test	7.6 (4.0–9.9)	9.1 (7.4–12.2)	NE	NE
*Sensitivity analysis* Cox PH model with no adjustment for covariates	NE	NE	0.86 (0.60–1.23)	NE
Sensitivity analysis Cox PH model with adjustment for treatment^d^	NE	NE	0.87 (0.61–1.25)	NE
*Sensitivity analysis* Cox PH model with adjustment for covariates identified by multivariate analyses^e^	NE	NE	0.85 (0.58–1.23)	NE

^a^Bayesian analyses values are credible intervals, frequentist analyses values are confidence intervals.

^b^The placebo plus gemcitabine arm had 48 events (deaths), censoring rate = 8%.

^c^The galunisertib plus gemcitabine arm had 84 events (deaths), censoring rate = 19%.

^d^From the Cox proportional hazards model including treatment and adjusted for the randomisation stratification factors: ECOG performance status, Disease stage (Investigator + Central Re-read) and previous gemcitabine treatment. P-values are based on the Wald test for the overall difference between the treatment/factor levels.

^e^Liver metastasis present at baseline, ECOG performance status, CA19-9 (normal, >ULN to <59 × ULN, ≥59 × ULN), post-discontinuation systemic anti-cancer therapy, previous gemcitabine treatment and sex were independent prognostic factors identified from stepwise Cox proportional hazards model for OS in multivariate analyses.

CA19-9 = carbohydrate antigen 19–9. *CI*=confidence interval; *CrI*=credible interval; *ECOG*=Eastern Cooperative Oncology Group; *HR*=hazard ratio; *NE*=not evaluable for given analysis method; *OS*=overall survival; *PH*=proportional hazards.

The median overall survival for the control group from the sensitivity Bayesian analysis with minimal borrowing of historical data for the control was higher and closer to the results using only data from this study. The median overall survival was 7.4 months (95% CrI: 5.7–10.0), the posterior median HR for overall survival was 0.83 (95% CrI: 0.59–1.19) (Table 1) and the posterior probability that the HR is <1 was 0.84, just <0.85. Frequentist sensitivity analyses using a Cox regression model adjusted for prognostic factors estimated the HR between the two treatment groups as 0.86, 95% CI: 0.60–1.23 (Fig. 1b, Table 1). No significant differences in progression-free survival were observed between the two treatments based on either investigator assessment or central readings (Fig. 1c, Supplementary Table S3). Previous treatment with gemcitabine was associated with improved overall survival (HR = 0.29, 95% CI: 0.13–0.63; data not shown).

The overall response and clinical benefit rates as measured by central read assessment were higher for the galunisertib group, but the differences were not statistically significant (Supplementary Table S3). Results of pain analyses are in Supplementary Fig. S3.

Investigator-determined Common Terminology Criteria for Adverse Events v 4.0 grade 3–4 events in phase 2 were mainly neutrophil count decreased, anaemia and fatigue (Table 2). In the galunisertib group vs. the placebo group, a higher percentage of patients had grade 3–4 neutrophil count decreased (36 [35%] of 103 vs. 14 [27%] of 52) and a slightly higher percentage of patients had grade 3–4 fatigue (13 [13%] of 103 vs. 5 [10%] of 52); conversely, a lower percentage of patients had grade 3–4 anaemia (12 [12%] of 103 vs. 9 [17%] of 52) and grade 3–4 platelet count decreased (8 [8%] of 103 vs. 6 [12%] of 52). There was a similar pattern for treatment-related grade 3–4 events (Supplementary Table S4). Few drug-related cardiac events were observed, and there was no significant difference in brain natriuretic peptide, cystatin C or ejection fraction between treatment groups. Thus, the cardiac safety was similar for both treatments. Likewise, no apparent difference in liver toxicity was observed (Supplementary Figure S4).

**Table 2:** Treatment-emergent adverse events (≥15% of any grade in phase 1b or phase 2 in either group) by CTCAE term

	Phase 1b			Phase 2^a^					
	Galunisertib + Gemcitabine (*N* = 14)			Placebo Group (*N* = 52)			Galunisertib Group (*N* = 103)		
	Grade 1–2	Grade 3	Grade 4	Grade 1–2	Grade 3	Grade 4	Grade 1–2	Grade 3	Grade 4
Anaemia	5 (36%)	3 (21%)	1 (7%)	19 (37%)	9 (17%)	0	32 (31%)	12 (12%)	0
Neutrophil count decreased	1 (7%)	4 (29%)	2 (14%)	7 (13%)	13 (25%)	1 (2%)	6 (6%)	33 (32%)	3 (3%)
Platelet count decreased	3 (21%)	2 (14%)	1 (7%)	14 (27%)	5 (10%)	1 (2%)	35 (34%)	8 (8%)	0
Nausea	10 (71%)	0	0	16 (31%)	2 (4%)	0	36 (35%)	5 (5%)	0
Vomiting	9 (64%)	0	0	16 (31%)	4 (8%)	0	22 (21%)	4 (4%)	2 (2%)
Constipation	5 (36%)	0	0	14 (27%)	2 (4%)	0	30 (29%)	1 (1%)	0
Abdominal pain	2 (14%)	2 (14%)	0	12 (23%)	3 (6%)	0	28 (27%)	8 (8%)	1 (1%)
Diarrhoea	5 (36%)	1 (7%)	0	12 (23%)	0	0	23 (22%)	0	1 (1%)
Fever	6 (43%)	0	0	11 (21%)	1 (2%)	0	36 (35%)	3 (3%)	0
Oedema limbs	4 (29%)	0	0	11 (21%)	1 (2%)	0	21 (20%)	2 (2%)	1 (1%)
Fatigue	7 (50%)	3 (21%)	0	21 (40%)	5 (10%)	0	41 (40%)	13 (13%)	0
ALT increased	4 (29%)	0	0	2 (4%)	1 (2%)	0	6 (6%)	5 (5%)	0
AST increased	4 (29%)	0	0	2 (4%)	1 (2%)	0	4 (4%)	4 (4%)	0
Anorexia	5 (36%)	0	0	12 (23%)	1 (2%)	0	26 (25%)	4 (4%)	1 (1%)
Hypocalcemia	3 (21%)	0	0	2 (4%)	0	0	4 (4%)	2 (2%)	0
Hypoalbuminemia	5 (36%)	0	0	1 (2%)	0	0	3 (3%)	0	1 (1%)
Headache	5 (36%)	0	0	6 (12%)	0	0	6 (6%)	0	0
Myalgia	4 (29%)	0	0	1 (2%)	0	0	7 (7%)	0	0

^a^In phase 2, Grade 5 events occurred that did not meet the ≥15% threshold; these included: (placebo group) 1 multiorgan failure, 1 peritoneal infection, 1 sepsis, and 1 ascites; (galunisertib group) 2 stroke, 1 pericardial effusion, 1 upper gastrointestinal haemorrhage, 1 infusion related reaction, 1 endocarditis infection, 1 lung infection, 1 arterial injury, and 1 respiratory failure.

*ALT*=alanine aminotransferase; *AST*=aspartate aminotransferase; *CTCAE*=Common Terminology Criteria for Adverse Event

Galunisertib was rapidly absorbed into the systemic circulation, reaching maximum concentrations typically within 1 h, and predicted exposures were within the therapeutic window (Supplementary Figure S5).

Baseline TGF-β1 levels were balanced between treatment groups (Supplementary Table S2). Estimates of the HR generally favoured patients with lower TGF-β1 at baseline (Supplementary Figure S6), suggesting that low TGF-β1 at baseline is predictive of response to galunisertib treatment (HR of 0.84 in patients with low TGF-β1 levels with an OS of 10.9 months for galunisertib group vs. 7.2 months for the placebo group). Patients in the galunisertib group with high TGF-β1 at baseline had worse survival compared with the placebo (Supplementary Figure S7).

Assessment of the relationship between TGF-β response and survival averaged across treatments (TGF-β responders vs. non-responders) gave a HR of 0.75 (95% CI: 0.52–1.10) (data not shown). For TGF-β responders, the median overall survival, pooled across treatments, was 10.1 months (95% CI: 8.2–12.7) vs. 6.7 months (95% CI: 3.6–10.2) for TGF-β non-responders. The percentage of patients achieving TGF-β1 reduction >20% was slightly higher in the galunisertib group (64 [69%] of 93) compared with the placebo group (30 [60%] of 50), with no significant difference in overall survival: 10.9 months (95% CI: 8.2–13.7) and 9.8 months (95% CI: 7.2–15.5), respectively (Supplementary Figure S8).

**Fig. 2:**
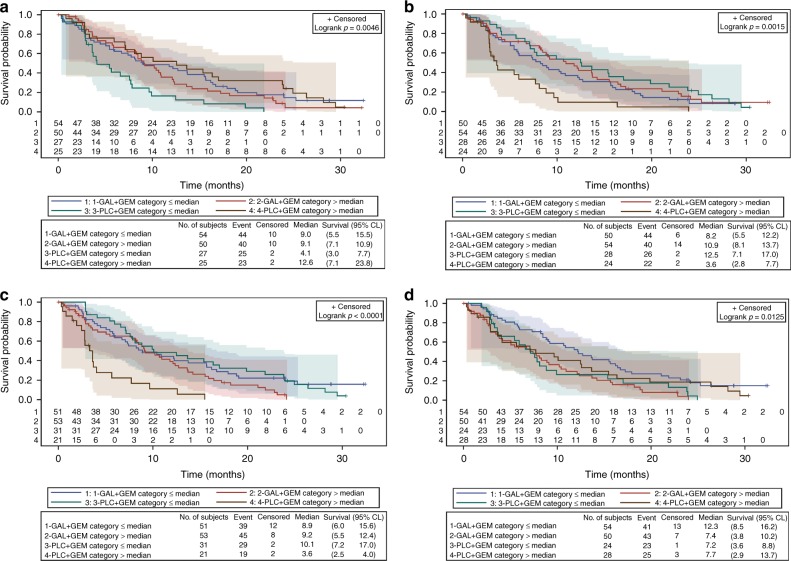
Kaplan–Meier estimates of survival by potential predictive factor, with 95% Hall–Wellner confidence bands and numbers at risk. Overall survival by plasma level of **a** follicle-stimulating hormone, **b** interferon-gamma induced protein 10, **c** macrophage inflammatory protein-1 alpha, and **d** plasminogen activator inhibitor 1. *CL*=confidence limits; *GAL*=galunisertib; *GEM*=gemcitabine; *No.*=number; *PLC*=placebo

After evaluating 279 plasma proteins from 156 patients, 249 proteins were evaluable for further examination. Of these, 31 proteins were identified as prognostic factors associated with survival (*p* < 0.001) across the study using the univariate Cox regressions model (Supplementary Figure S9). Additionally, 4 proteins were identified to be predictive factors associated with survival (unadjusted for other prognostic variables) following galunisertib treatment: interferon-gamma induced protein 10 (IP-10) (>median at baseline HR = 0.40, 95% CI: 0.23– 0.67; ≤median at baseline HR = 1.35, 95% CI: 0.83–2.21), follicle-stimulating hormone (>median at baseline HR = 1.40, 95% CI: 0.83–2.37; ≤median at baseline HR 0.45, 95% CI: 0.27–0.75), macrophage inflammatory protein-1 α (MIP-1α) (>median at baseline HR = 0.38, 95% CI: 0.22–0.66; ≤median at baseline HR = 1.02, 95% CI: 0.63–1.65) and plasminogen activator inhibitor 1 (>median at baseline HR = 1.45, 95% CI: 0.88–2.41; ≤median at baseline HR = 0.55, 95% CI: 0.33–0.92) (*p* = 0.01) (Fig. 2). The levels of these proteins did not change over time post-treatment in either group.

T-cell counts were reduced in both arms over the course of the study; however, patients who had >50% decrease in T cells from baseline in the first three cycles of treatment had worse overall survival (Supplementary Figure S10).

## Discussion

The present study is the first randomised study of galunisertib in pancreatic cancer. We used a randomisation favouring the experimental group (2:1) and a pre-defined Bayesian-augmented design to enrich the control group data with pre-existing historical data. Using this approach, the novel combination of galunisertib plus gemcitabine showed an improvement in survival over gemcitabine alone. Considering the study design, the type I error of 0.16 may be considered high relative to other phase 2 studies; however, the primary analysis method exceeded the threshold probability (*p*) required for the study to be positive quite substantially (*p* [HR < 1] = 0.93, whereas 0.85 was required to pass). Because this Bayesian-augmented design is rare in phase 2 studies, we performed sensitivity analyses to determine whether unexpected bias was introduced, including a frequentist analysis using only data from the study. In these supporting sensitivity analyses, the HR ranged from 0.83 to 0.87, which still favours galunisertib treatment. Additionally, all known prognostic factors were well-balanced across treatment arms and therefore, minimal bias was introduced into the study by the 2:1 randomisation. None of the survival analyses were post hoc, but were all pre-specified in order to legitimately consider the totality of evidence from phase 2 study results when making decisions on future development. However, it should also be noted that the power of the study design was reduced when using the study data alone.

Over the past decades and at the time this study started, gemcitabine was considered the reference treatment in advanced pancreatic cancer.^10^ Only recently, the combination of gemcitabine with the new agent nab-paclitaxel^14^ or the three-drug combination regimen FOLFIRINOX^11^ were able to provide a survival improvement over gemcitabine single agent. However, these regimens are significantly more toxic than gemcitabine and are recommended for patients who have an ECOG performance status 0–1,^12^ whereas gemcitabine monotherapy remains recommended for >40% of patients who have either an ECOG PS 2^13^ or a comorbidity profile that precludes more aggressive regimens. Therefore, there remains a clear unmet need for the development of gemcitabine-based regimens that are efficacious and more tolerable than combination chemotherapy options. Considering the overall safety profile of galunisertib plus gemcitabine did not significantly differ from gemcitabine alone, the present findings suggest that galunisertib plus gemcitabine may be an attractive alternative option, especially for frail patients who are unable to tolerate standard chemotherapy combination regimens. The positive efficacy signals for galunisertib from this present study and its role in modulating the TGF-β pathway have prompted interest in future validation of the efficacy of galunisertib plus gemcitabine as well as ongoing clinical trials of galunisertib with immunotherapeutic agents (anti-PD-L1 mAb durvalumab, NCT02734160). The intentions of these trials are to generate evidence for development of TGF-β pathway inhibitors in combination with more modern therapeutic approaches.

Pancreatic cancer is characterised by a highly immunosuppressive tumour microenvironment that aids rather than controls cancer development and progression,^15^ and TGF-β is commonly viewed as the most powerful immunosuppressive cytokine.^16^ In a recently integrated genomic analysis of pancreatic cancer, programmes enriched with a macrophage or T-cell co-inhibition specific signature co-segregated with poor survival.^7^ Additionally, we recently demonstrated that high plasma levels of cytokines involved in macrophage recruitment, including MIP-1α, or FOXP3^+^ regulatory T cells (T_reg_) enrichment, including IP-10, were negatively associated with pancreatic cancer patients’ survival.^17^ Thus, we examined markers associated with inflammatory and other immunologic processes in pancreatic cancer to potentially select patients who may benefit from TGF-β receptor inhibition. Indeed, we identified MIP-1α and IP-10 as two of the four most significant predictive factors for the efficacy of galunisertib plus gemcitabine. We confirmed that patients with high circulating levels of MIP-1α or IP-10 in the control group had a significantly shorter overall survival. Populations with such a poor prognosis received the greater benefit by the combination treatment with galunisertib. T-cell subsets were not different between the arms and patients with <50% reduction in T cells had a better overall survival than those with greater reductions, a relationship that may be linked to chemotherapy. Further studies addressing the effects of targeting TGF-β signalling by galunisertib in macrophages and T_reg_ cells, and the consequent potential modulation of the immunosuppressive tumour microenvironment and chemoresistance in pancreatic cancer are warranted.

The pattern of treatment effect in the two TGF-β responder groups was different. Within the responder group, median overall survival was similar across the treatments; however, median overall survival was improved for galunisertib plus gemcitabine in the non-responder group. Distribution of potential prognostic factors between the responder groups and treatment arms is under investigation, but this study was too small to ascertain possible reasons for the observation. Nonetheless, the association of reduced TGF-β1 levels with improved survival has been observed in another disease,^18^ suggesting that reductions in TGF-β1 levels could represent an additional tool to assess clinical benefit even if it may not be specific to a TGF-β inhibitor.

In conclusion, the present study indicates a positive signal of efficacy for the combination of galunisertib and gemcitabine in improving overall survival vs. gemcitabine in patients with unresectable pancreatic cancer, with minimal added toxicity. Biomarker analyses provide evidence of patient subgroups with higher levels of cytokines recruiting macrophages or regulatory T cells that may benefit to a greater extent from treatment with galunisertib plus gemcitabine. Collectively, this evidence warrants further clinical development for galunisertib in combination with more modern chemotherapeutic or immunotherapeutic agents for patients with unresectable pancreatic cancer. One trial evaluating galunisertib in combination with durvalumab in pancreatic cancer is ongoing (NCT02734160).

## Electronic supplementary material

Supplementary Materials

Supplementary Figures

Supplementary Tables

## Availability of data and materials

All data generated during the study is available at Eli Lilly.

## PREVIOUS PRESENTATION

The data from this study were presented in part at ASCO 2013, AACR2016 and ASCO 2016.
