# Supplementary material for: Galunisertib plus gemcitabine vs. gemcitabine for first-line treatment of patients with unresectable pancreatic cancer
Source: Br J Cancer. 2018 Oct 15;119(10):1208–14. doi: 10.1038/s41416-018-0246-z (PMC6251034; doi:10.1038/s41416-018-0246-z)
Supplement: Supplementary file 1 — Supplementary Materials [file 41416_2018_246_MOESM1_ESM.docx]

**Supplementary Materials**

**Supplementary Methods**

***Study Design and Participants***

Dose escalation of galunisertib (plus a standard dose of gemcitabine) occurred in cohorts of 3–6 evaluable patients until at least two patients per cohort experienced a dose-limiting toxicity or 300 mg/day dose of galunisertib was reached. In the absence of dose-limiting toxicities and before reaching the maximum dose, dose escalation could have been stopped if a predetermined pharmacokinetic endpoint was reached.^1^ In phase 2, patients were given galunisertib plus gemcitabine (galunisertib group) or placebo plus gemcitabine (placebo group). The study was conducted according to the principles of good clinical practice, applicable laws and regulations, the Council for International Organizations of Medical Sciences International Ethical Guidelines, and the Declaration of Helsinki. Each institution’s ethical review board approved the study. All patients signed an informed consent document before study participation.

Phase 1b entry required histological or cytological evidence of a diagnosis of cancer that was advanced and/or metastatic disease, that was refractory to standard therapy and/or therapies known to provide clinical benefit or for which no standard therapy exists, and in which gemcitabine therapy at the proposed doses and schedule would have been considered appropriate treatment for the metastatic disease. Patients were included with measurable disease or non-measurable disease, defined according to Response Evaluation Criteria in Solid Tumors guidelines version 1.1 (RECIST, v. 1.1). Patients may have received prior chemotherapy, radiotherapy, cancer-related hormone therapy, or other investigational therapy as treatment. There was no limit to the number of previous lines of therapy.

Phase 2 entry required: histological or cytological diagnosis of adenocarcinoma of the pancreas that was locally advanced (stage II, III) or metastatic (stage IV) and not amenable to resection with curative intent (for stage II patients to be included: patients had severe comorbidities or advanced age); post-surgical progression after previous radical surgery for pancreatic cancer; ≥3 months since previous adjuvant chemotherapy or chemoradiotherapy with gemcitabine or other commonly used cytotoxic agents; and measurable disease or non-measurable disease, defined according to RECIST v. 1.1. Patients with endocrine pancreatic tumours or ampullary cancer were excluded.

Inclusion in either study phase required the patients to be ≥18 years of age; have adequate hematologic, hepatic, and renal function; have a performance status of ≤2 on the Eastern Cooperative Oncology Group (ECOG) scale; and have recovered from any grade 3–4 toxicities of previous therapies. Exclusion criteria included moderate or severe cardiac disease.

***Bayesian Exponential-Likelihood Model***

The primary objective of phase 2 was to compare overall survival between galunisertib plus gemcitabine (galunisertib group) and placebo plus gemcitabine (control group) using a Bayesian augmented control design. Bayesian designs can help to leverage pre-existing information for the control group, enabling a reduced sample size for this treatment group and increased exposure via a 2:1 randomisation ratio to the experimental treatment. Patient-level historical data from two previous randomised trials^2,3^ (Supplementary Table S1) that included gemcitabine were utilized for the Bayesian analysis of overall survival. We used the survival data from the combined sample of 330 patients who received study drug (66 from Saif and colleagues^3^; 264 from Oettle and colleagues^2^) with newly diagnosed pancreatic cancer who are considered to be from a similar population as those in the current study. The Kaplan-Meier estimates of median overall survival from the two studies are 8.3 (95% CI: 6.5–10.8) and 6.3 (95% CI: 5.4–6.9) months, respectively. These estimates are considered comparable to more recent gemcitabine data^4^ reporting median overall survival of 6.7 (95% CI: 6.0–7.2) months, further justifying the use of Bayesian analysis in this current study.

The primary method of analysis for comparing overall survival between the treatment groups used a Bayesian exponential-likelihood model with a hierarchical random-effects distribution on treatment effects. If the Bayesian posterior probability of superiority of the experimental treatment over control (that is, hazard ratio [HR] <1) exceeded 0.85, then it was to be concluded that the experimental arm was superior. The amount of information from the historical controls incorporated by the Bayesian-augmented control model depends on both the strength of the a priori belief of the similarity of the hazard rates between the historical controls and the current control arm, as well as the estimated similarity once the data are analysed. If the hazard rates are similar, more historical control information is “borrowed” than in scenarios where the rates differ.

In the Bayesian analysis, the degree of borrowing is governed by the model parameter $\tau.$ This parameter is the standard deviation of the log (HR) of the assumed population of historical studies relative to the current control arm. If the data in the current control arm are concordant with the historical data, then the estimates of $\tau$ will be small and there will be a larger amount of borrowing from the historical studies. Conversely, if the data from the current control arm are dissimilar to the historical studies, then the estimates of $\tau$ will be large, and less information is borrowed.

***Procedures***

Tumour burden and anatomical lesion locations were assessed based on RECIST v. 1.1. An Independent Review Committee conducted a blinded assessment of the medical imaging data using the available computed tomography (CT) or magnetic resonance imaging (MRI) for each patient at baseline and at each subsequent time point to determine the date of disease progression and the best overall response.

The safety population consisted of patients who received at least one dose of study drug. Safety monitoring was conducted throughout the study and for 30 days after the last dose of investigational product or, in the case of ongoing drug-related toxicity, until resolution. Adverse events were assessed using Common Terminology Criteria for Adverse Events (CTCAE) v 4.0. The nature and severity of adverse events, relatedness to study drug, dose-limiting toxicities, and laboratory changes were evaluated. In addition, cardiac safety was assessed by echocardiography/Doppler, chest CT/MRI, serial measurements of brain natriuretic peptide, troponin I, cystatin C, and high-sensitivity C-reactive protein.

***Pharmacokinetics Methods***

Pharmacokinetic and pharmacodynamic blood sampling were matched to evaluate exposure-associated and biomarker changes. Human plasma samples obtained during this study were analysed for galunisertib using validated liquid chromatography–mass spectrometry/mass spectrometry methods (BPLY215A and BPLY215B). The lower limit of quantification was 0.050 ng/mL and the upper limit of quantification was 10.000 ng/mL for BPLY215A. The lower limit of quantification was 5.000 ng/mL and the upper limit of quantification was 1000.000 ng/mL for BPLY215B. Patient demographic factors potentially influencing galunisertib disposition included age, body weight, body mass index at baseline, sex, alcohol, smoking, and caffeine use.

***Biomarker and Pharmacodynamics Methods***

Potential clinical prognostic and predictive factors including levels of circulating carbohydrate antigen 19-9 (CA19-9) and transforming growth factor-beta (TGF-β) measured at baseline were evaluated for their impact on overall survival. In circulating blood, (CA19-9) kinetics were evaluated using standard laboratory testing (Q2 Solutions, Morrisville, NC). Plasma samples from patients were analyzed for levels by enzyme‑linked immunosorbent assay (ELISA) (R&D Systems, DB100B, Minneapolis, MN). Levels of CA19-9 and TGF-β were only analysed during the first 12 weeks of treatment. Patients were considered a CA19-9 responder if they had a reduction of >20% in CA19-9 levels in the first 12 weeks of treatment, in contrast to 8 weeks reported in other studies.^5^ Patients with >20% reduction in TGF-β levels in the first 12 weeks were defined as TGF-β responders. CA19-9 response patterns were compared to the reduction of TGF-β levels in the same responder population. Overall survival was summarized descriptively by treatment group and response status using the Kaplan-Meier method.

Platelet factor 4 (PF4) levels were assessed to determine possible platelet activation although it was expected that pancreatic patients will have elevated PF4 levels as part of their tumour-associated intravascular coagulopathy.

Continuous lab variables were categorized according to quartile values calculated across all patients (≤lower quartile [LQ], >LQ to ≤median, >median to ≤upper quartile [UQ], >UQ). Additional categorizations of TGF-β were generated relative to the normal range (within normal limits, >upper limit of normal [ULN] to <59×ULN, ≥59×ULN) and based on a galunisertib study in hepatocellular carcinoma patients showing improved overall survival among TGF-β responders (≥20% reduction) and higher response rates among those with baseline TGF-β ≥3411 pg/mL^6^ (≤median vs >median and <3400 pg/mL vs ≥3400 pg/mL). Univariate Cox proportional hazards models were first used to select covariates with p≤0.2. For these covariates, a multivariate Cox model was used to make stepwise selection with both entry and exit p=0.2 in order to identify independent prognostic factors.

Potential predictive markers, similarly split into two groups at the median, were evaluated to determine if baseline levels were predictive for a treatment response to galunisertib based on overall survival. Cox models were used with terms including the interaction of baseline protein marker (>median, ≤median) and treatment arm (galunisertib plus gemcitabine, gemcitabine alone), and a marker was identified to be potentially predictive if the interaction term had a p≤0.05. There was no adjustment for multiplicity.

Standard flow cytometry was used to investigate T cell subsets, and CD3 levels were assayed by using a test based on epigenetic alterations.^7^ Analyses were conducted on 279 circulating proteins whose concentrations were estimated by Myriad RBM (Austin, TX, USA) using the Human Multi-Analyte Profiles (MAP®) panel. Whole blood samples were used to determine the levels of T cell subsets such as CD4^+^ (normal range=441–2156 cells/UL), CD8^+^ (normal range=125–1312 cells/UL) and CD4^+^CD25^+^CD127^-^/LOlP3^+^ (normal range estimated to be 18–86 cells/UL) by standard flow cytometry. In addition, percentage of FOXP3 (normal range=1–3.6%) and CD3 (normal range=17–36%) were determined in whole blood using an epigenetic T cell assay (Epiontis, Berlin, Germany).^6^

*Exploratory Analyses*

Approximately 30 markers of the roughly 279 multiplasma proteins assessed via MAP had >50% of samples below the limit of quantification at baseline and were excluded from analysis. Baseline concentrations of the remaining proteins were evaluated for their impact on overall survival to identify if any could be investigated further as potential prognostic and/or predictive markers. Each parameter was split into two groups at the median for >median versus ≤median comparison. Univariate Cox models were used to select potentially prognostic markers where the effect of the marker was significant at p≤0.001. Multi-variate Cox models with terms including the baseline of the marker (>median, ≤median), treatment, and their interaction were used to identify potentially predictive markers based on whether the interaction term p≤0.01. There was no adjustment for multiplicity.

Changes in T cells compartment (CD4+, CD8+, T_regs_, and CD3+) post-treatment were evaluated by mixed effect model repeated measures. Data were log-transformed prior to analysis and the ratio to baseline evaluated, with baseline included as a covariate, and fixed effect terms of treatment, visit, and the interaction of treatment and visit. Overall survival was evaluated for patients who had ≤50% decreases in T cells from baseline and by treatment group using the Kaplan-Meier method.

**Supplementary Results**

***Phase 1 Results***

Of the 18 patients who entered screening for phase 1b of the study, four were screen-failures (Supplementary Fig. S1). Galunisertib was administered at 80 mg/day (n=5), 160 mg/day (n=4), and 300 mg/day (n=5). In the phase 1b part, most patients had gastrointestinal tumours, including four patients with pancreatic cancer (Supplementary Table S2). Galunisertib treatment was well tolerated (Table 2) and 300 mg/day was identified as the phase 2 dose.

A median of 2.5 (range=0–9) and 1.0 (range=0–8) cycles were completed per patient for galunisertib and gemcitabine, respectively. Dose omissions occurred for galunisertib in 5 patients and for gemcitabine in 7 patients; dose reductions occurred for gemcitabine in 9 patients; dose discontinuations occurred for galunisertib for 3 patients; and dose delays occurred for gemcitabine in 6 patients. A total of 12 patients died during this phase (80 mg/day: n=5, 160 mg/day: n=3, 300 mg/day: n=4). With a manageable toxicity profile, signals of anti-tumour activity in the pancreatic cancer patients (Table 2), and no dose-limiting toxicities in phase 1b, 300 mg/day was identified as the phase 2 dose.

***Overall Survival Analyses***

The primary Bayesian analysis used an Inverse Gamma (0.5, 200) distribution for $\tau^{2}$. This distribution is approximately centered at 0.1 with an equivalent weight of n=1. Given that the observed control group in this study was comparable with historical controls, in the final analysis the Bayesian augmented control model estimate of $\tau$ was relatively small, and the model borrowed information with weight equating to approximately 37 events from the historical studies.

Because this pre-defined Bayesian design is a relatively new approach for assessing anti-tumour activity in early phase studies, we conducted a number of sensitivity evaluations to understand better the robustness of the study conclusions and the value of this approach. This secondary evaluation included a Bayesian model with weak borrowing, adjustment for the randomisation factors (Eastern Cooperative Oncology Group [ECOG] status, disease stage, previous gemcitabine treatment) and other clinical prognostic factors, and descriptive or frequentist analysis of overall survival, including Kaplan-Meier method and Cox proportional hazards models. The Kaplan-Meier estimate of median survival time was 9.1 months (95% CI: 7.4–12.2) in the galunisertib group compared with 7.6 months (95% CI: 4.0–9.9) in the placebo group, log rank test p=0.4014. Across the various sensitivity analyses of overall survival, the HR ranged from 0.79 (95% CI: 0.59–1.09) to 0.87 (95% CI: 0.61–1.25) (Table 1).

Several clinical characteristics were found to be prognostic for overall survival (Supplementary Fig. S6). Liver metastasis present at baseline, ECOG performance status, CA19-9 (>upper limit of normal [ULN] to <59×ULN, ≥59×ULN), post-discontinuation systemic anticancer therapy, previous gemcitabine treatment and sex were independent prognostic factors for overall survival in multivariate analyses. Adjustment for the significant prognostic factors in Cox proportional hazards models for overall survival was concordant with results from other sensitivity analyses of overall survival (Table 1).

***Population Pharmacokinetics***

Galunisertib concentrations were determined from all evaluable patients. Galunisertib was rapidly absorbed into the systemic circulation, reaching maximum concentrations typically within 1 hour (Supplementary Fig. S5). The calculated median (25th–75th) population exposure at steady state was 5.56 mg*h/L (95% CI: 3.82–7.91) with maximum concentration (C_max_) and time to maximum concentration (t_max_) of 904 ng/mL (95% CI: 668–1194) and 1.5 h (95% CI: 1–2.5), respectively. Although there was one patient with very high exposure, the predicted exposures at steady state (AUC_0-24,ss_) following 300 mg/day dose, administered as 150 mg BID, were within the therapeutic window (3730 to 8380 ng*hr/mL) defined previously.^8^ The mean (% standard error of estimation) apparent population clearance (CL/F) and volume of distribution of galunisertib were 35 (3) and 190 (18), respectively, with between-patient variability on apparent clearance of 47%. There was a small but statistically significant effect of age on CL/F.

***Health Outcomes***

Patient-reported symptoms, such as patient-reported pain using the Brief Pain Inventory short form (BPI-sf)^9^ and investigator-rated analgesic level were evaluated in phase 2 only. The BPI-sf questionnaire is a 9-item patient-reported instrument used as a multiple-item measure of cancer pain intensity and was administered to the patient prior to randomisation and prior to the start of each cycle (before study treatment administration) as well as at follow up (approximately 30 days after the last dose of study treatment).

The majority compliance of BPI-sf administration ranged from approximately 70–90% across visits. At baseline, the mean pain score (standard deviation [SD]) was 2.48 (2.21) for the placebo group and 2.60 (2.06) for the galunisertib group. There were no differences between the two treatment groups post-treatment (Supplementary Fig. S3). Mean pain (SD) interference scores at baseline were 3.20 (2.78) for the placebo group and 2.83 (2.46) for the galunisertib group. Pain interference scores were reduced in both treatment groups up to Cycle 6 (data not shown). Finally, analgesia intake was similar between both treatment groups, which included drugs such as aspirin, paracetamol, nonsteroidal anti-inflammatory drugs, codeine, dextropropoxyphene, pentazocine, oxycodone, and hydrocodone. In both treatment groups, a reduction from baseline in analgesic consumption levels was observed across the post-baseline cycles up to Cycle 13 and, as would be expected, levels increased at the follow-up visit when patients were experiencing more pain (data not shown).

**References**

1. Herbertz S, Sawyer JS, Stauber AJ, et al. Clinical development of galunisertib (LY2157299 monohydrate), a small molecule inhibitor of transforming growth factor-beta signaling pathway. *Drug Des Devel Ther* 2015;9:4479-4499.
2. Oettle H, Richards D, Ramanathan RK, et al. A phase III trial of pemetrexed plus gemcitabine versus gemcitabine in patients with unresectable or metastatic pancreatic cancer. *Ann Oncol* 2005;16(10):1639-1645.
3. Saif MW, Oettle H, Vervenne WL, et al. Randomized double-blind phase II trial comparing gemcitabine plus LY293111 versus gemcitabine plus placebo in advanced adenocarcinoma of the pancreas. *Cancer J* 2009;15(4):339-343.
4. Von Hoff DD, Ervin T, Arena FP, et al. Increased survival in pancreatic cancer with nab-paclitaxel plus gemcitabine. *N Engl J Med* 2013;369(18):1691-1703.
5. Sideras K, Braat H, Kwekkeboom J, et al. Role of the immune system in pancreatic cancer progression and immune modulating treatment strategies. *Cancer Treat Rev* 2014;40(4):513-522.
6. Travis MA, Sheppard D. TGF-β activation and function in immunity. *Annu Rev Immunol* 2014;32:51-82.
7. Baron U, Floess S, Wieczorek G, et al. DNA demethylation in the human FOXP3 locus discriminates regulatory T cells from activated FOXP3(+) conventional T cells. *Eur J Immunol* 2007;37(9):2378-2389.
8. Gueorguieva I, Cleverly AL, Stauber A, et al. Defining a therapeutic window for the novel TGF-β inhibitor LY2157299 monohydrate based on a pharmacokinetic/pharmacodynamic model. *Br J Clin Pharmacol* 2014;77(5):796-807.
9. Cleeland CS. Pain assessment in cancer. In: Osaba D, ed. *Effect of Cancer on Quality of Life*. Boca Raton, FL: CRC Press; 1991:293-305.
