# Supplementary material for: Galunisertib plus gemcitabine vs. gemcitabine for first-line treatment of patients with unresectable pancreatic cancer
Source: Br J Cancer. 2018 Oct 15;119(10):1208–14. doi: 10.1038/s41416-018-0246-z (PMC6251034; doi:10.1038/s41416-018-0246-z)
Supplement: Supplementary file 2 — Supplementary Figures [file 41416_2018_246_MOESM2_ESM.docx]

**
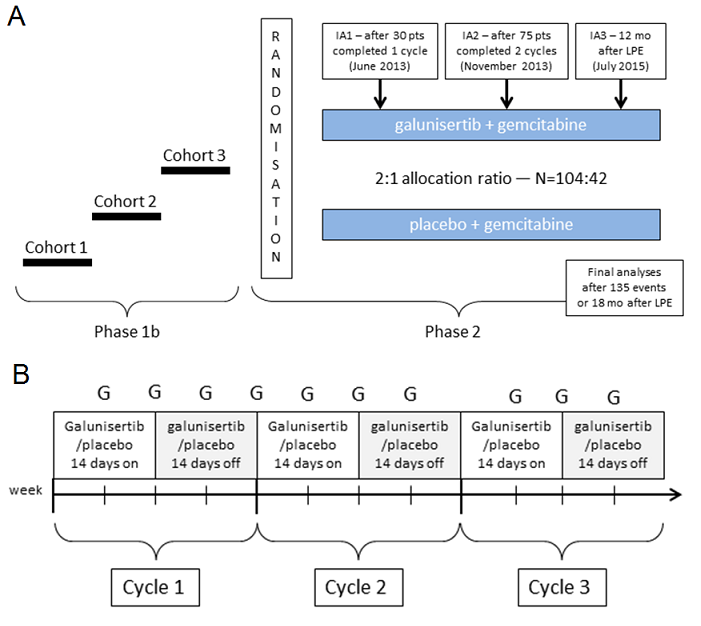
**

**Fig. S1. Trial design.** (A) Treatment flow and analysis. (B) Dosing. IA=interim analysis; G=gemcitabine; Galu=galunisertib; LPE=last patient enrolled; mo=months; pts=patients

**
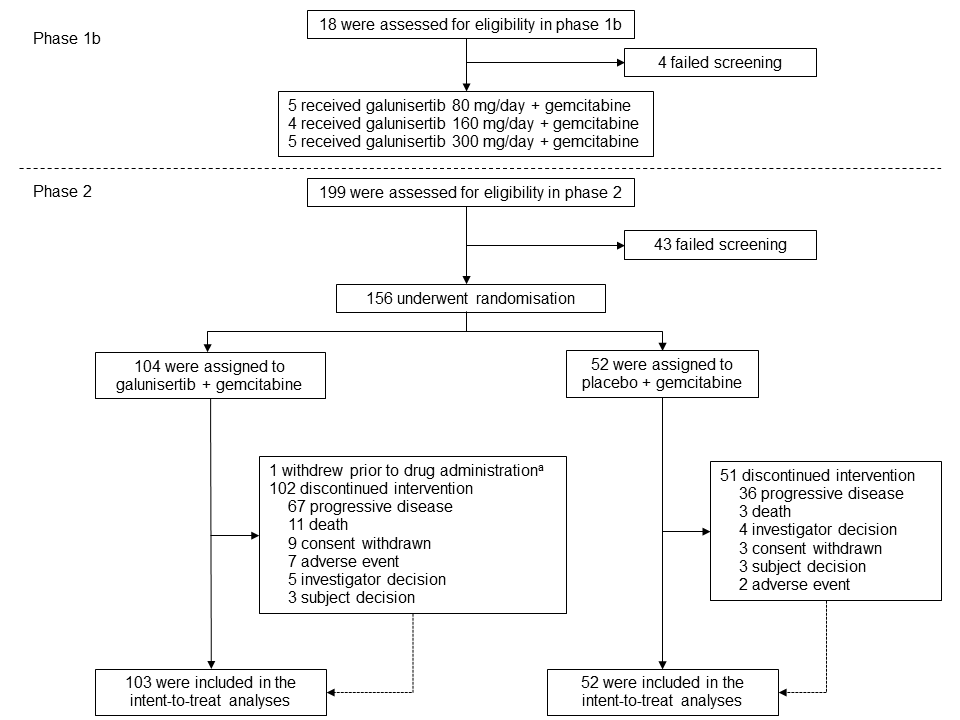
**

**Fig. S2. Trial profile.** Note: One patient was enrolled but withdrew prior to study drug administration; therefore, the galunisertib group included 103 patients.


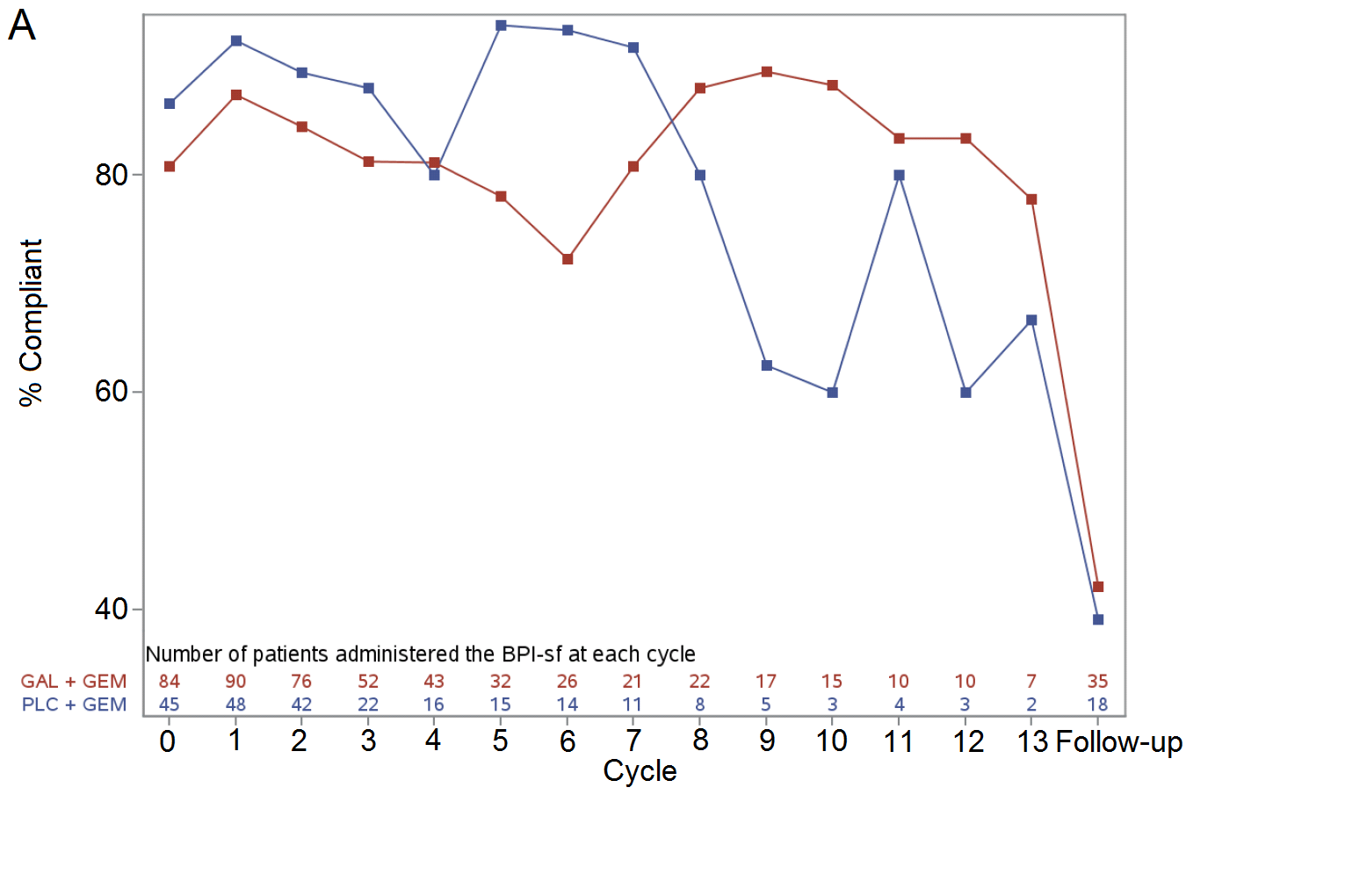


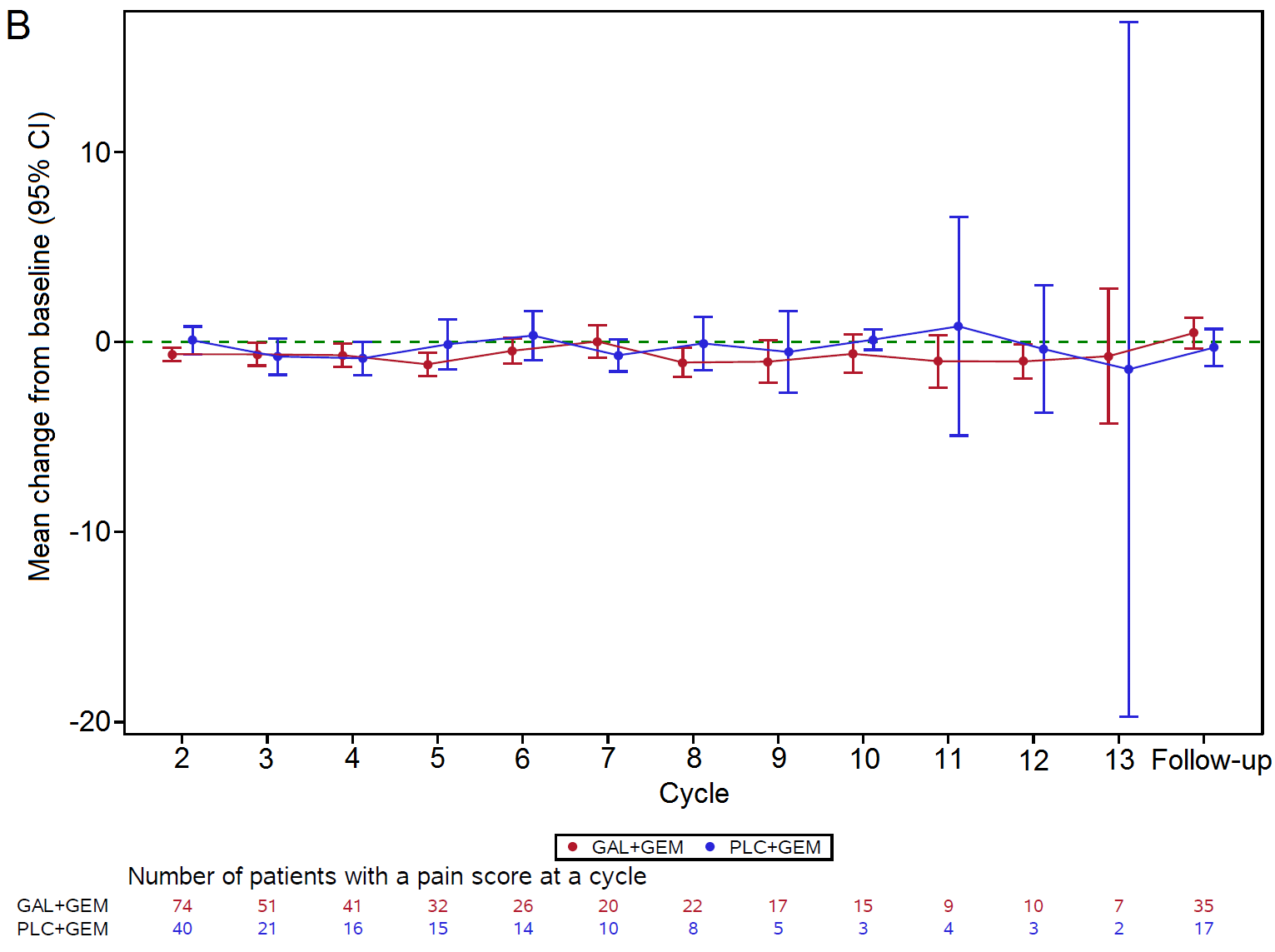


**Fig. S3. Brief Pain Index.** (A) Brief Pain Index – short form compliance. (B) Mean pain score. Baseline is the average of the values from the questionnaires administered at screening and pre-dose at cycle 1. BPI-sf=Brief Pain Inventory – short form; CI=confidence interval; GAL=galunisertib; GEM=gemcitabine; PLC=placebo


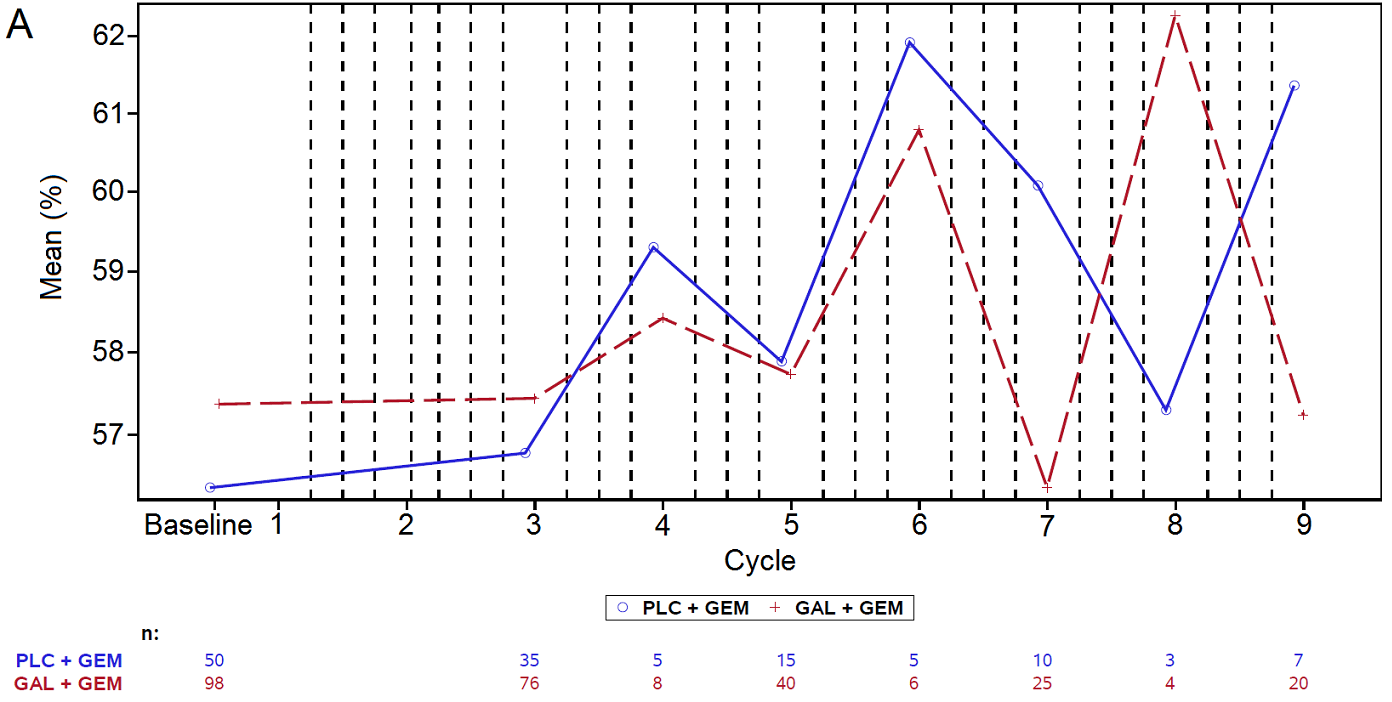


**
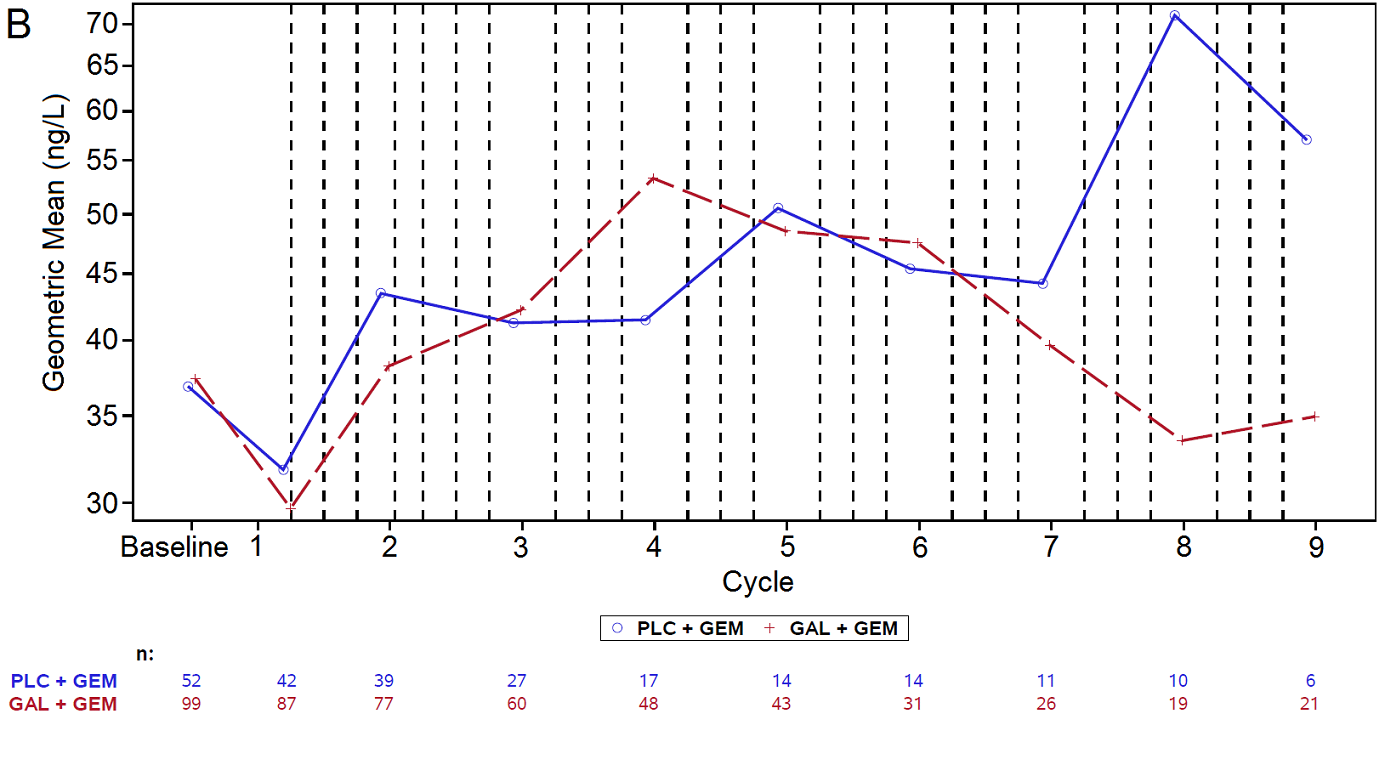
**


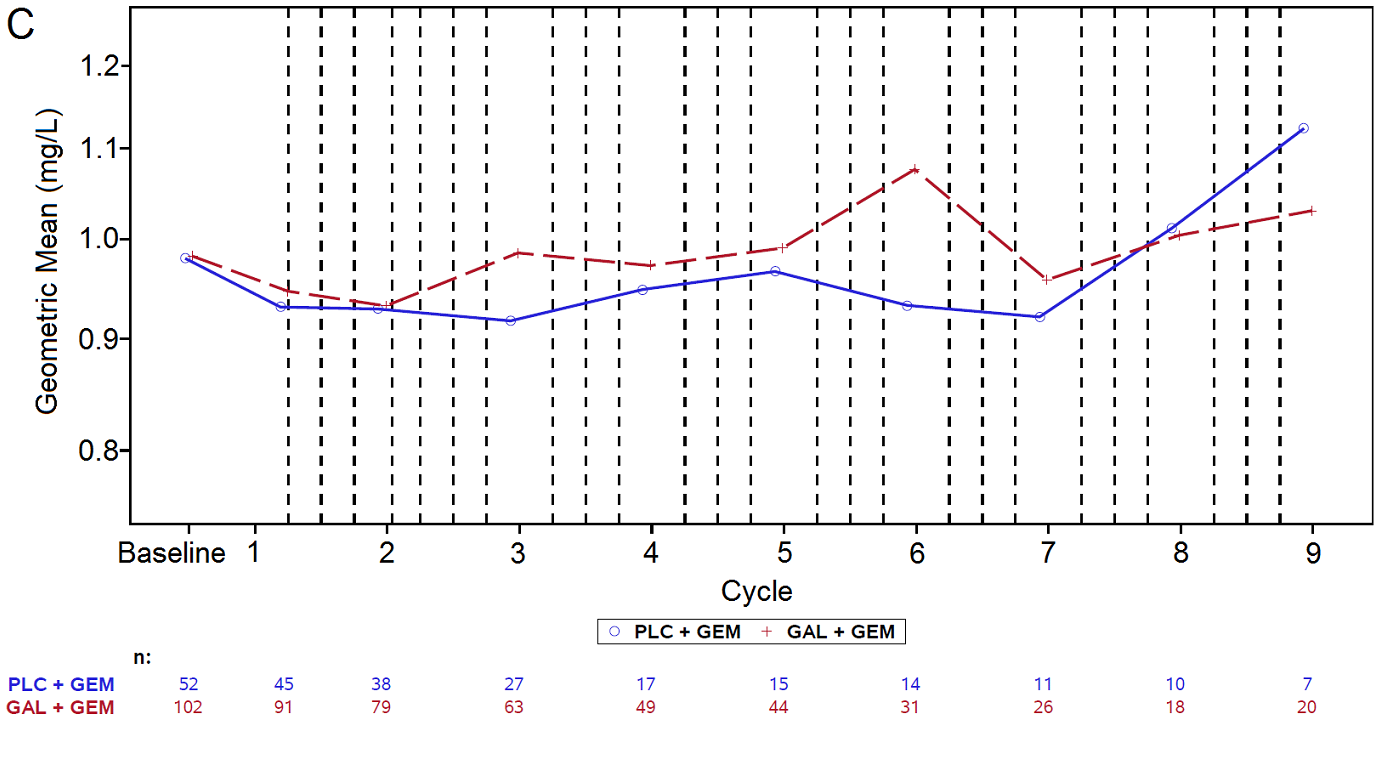


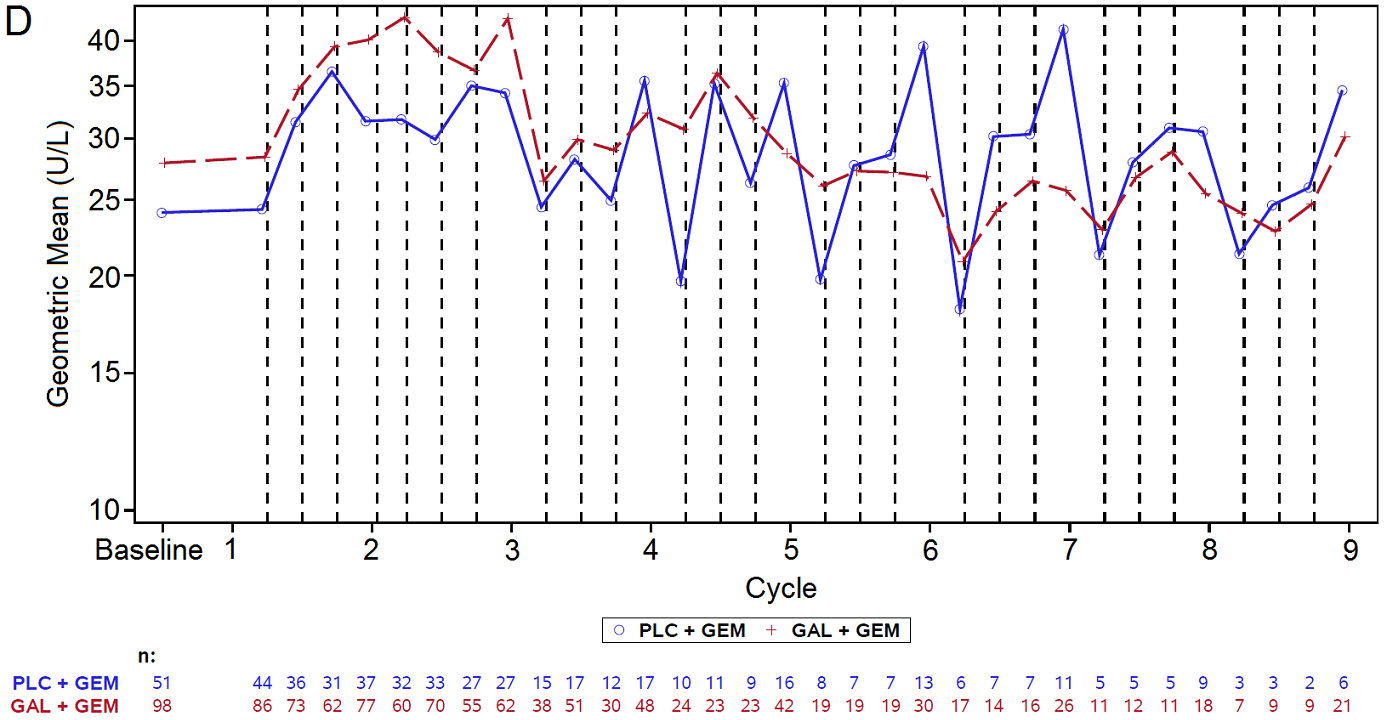


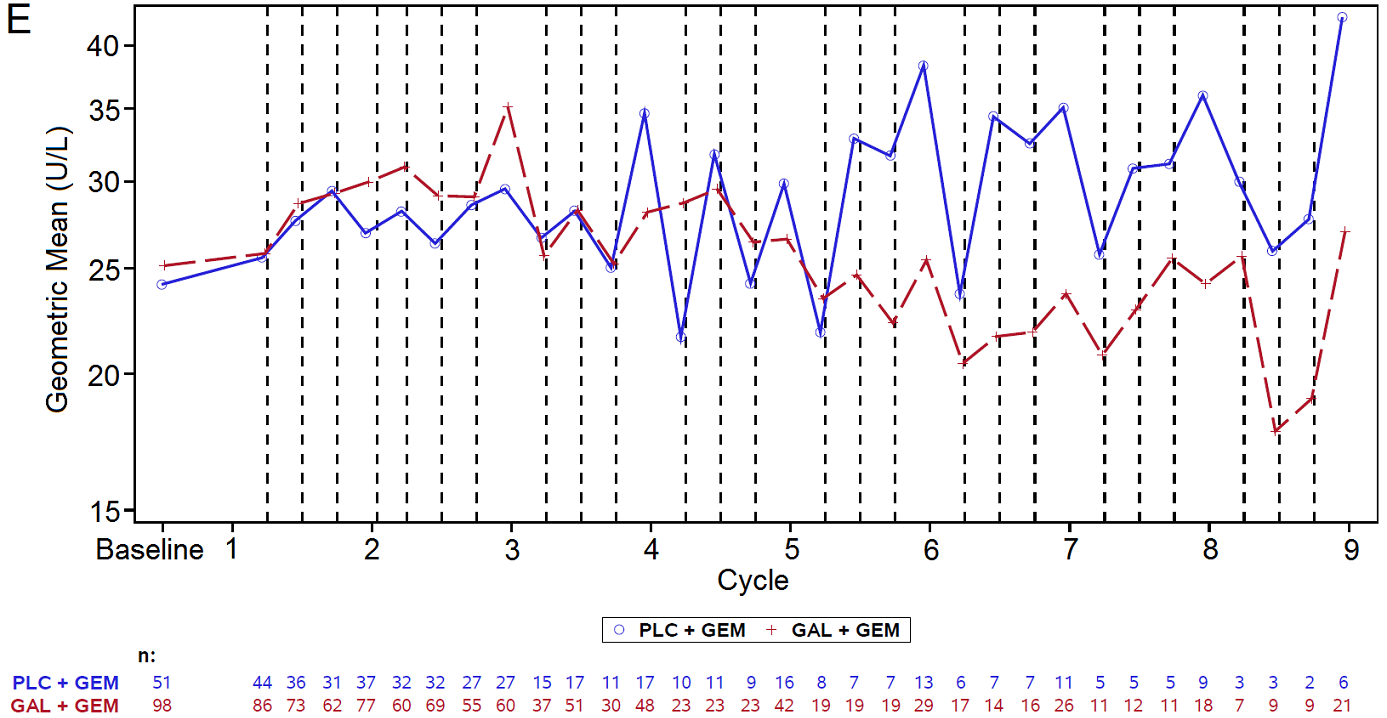


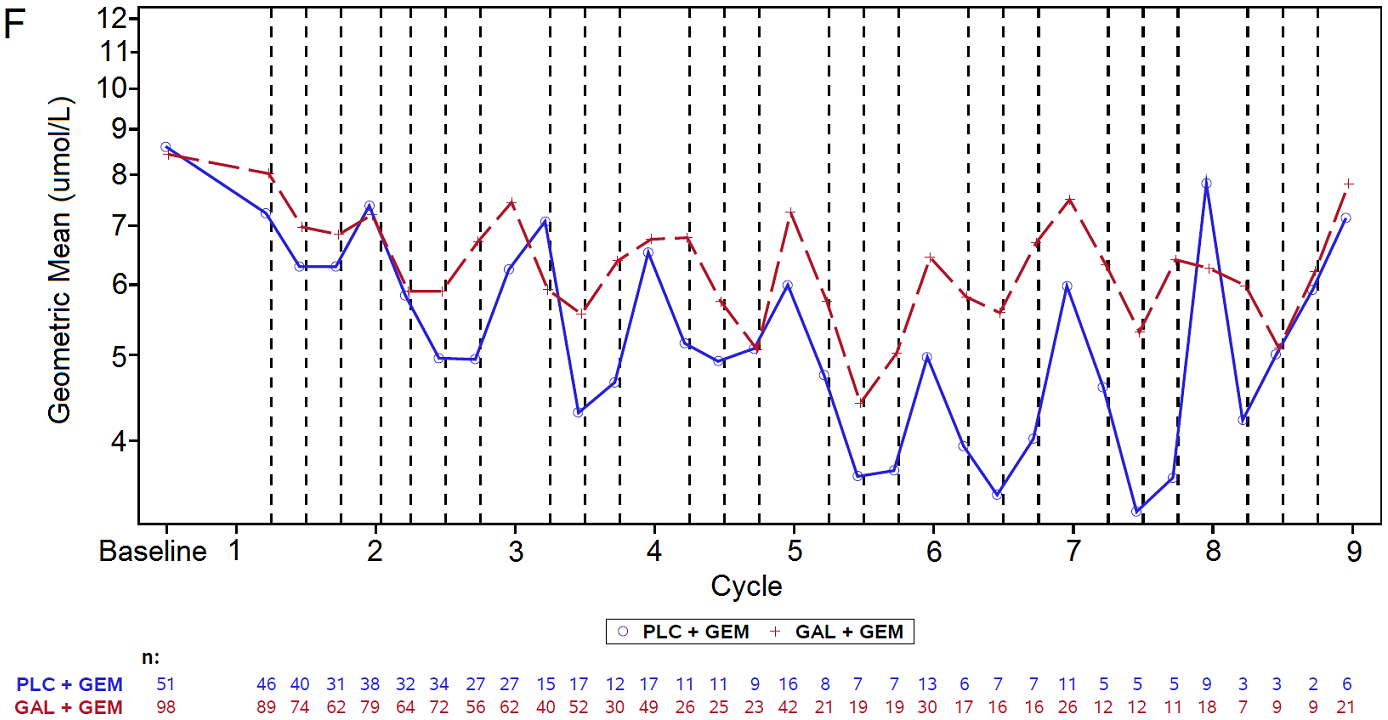


**Fig. S4. Cardiac and liver function assessments.** (A) Left ventricular ejection fraction. (B) Brain natriuretic peptide. (C) Cycstatin C. (D) Alanine aminotransferase. (E) Aspartate aminotransferase. (F) Bilirubin. GAL=galunisertib; GEM=gemcitabine; PLC=placebo


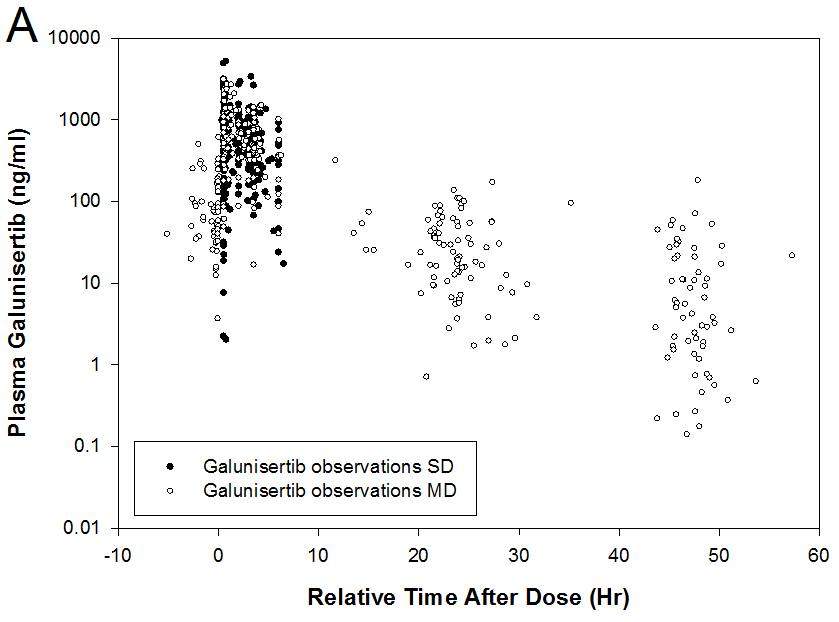


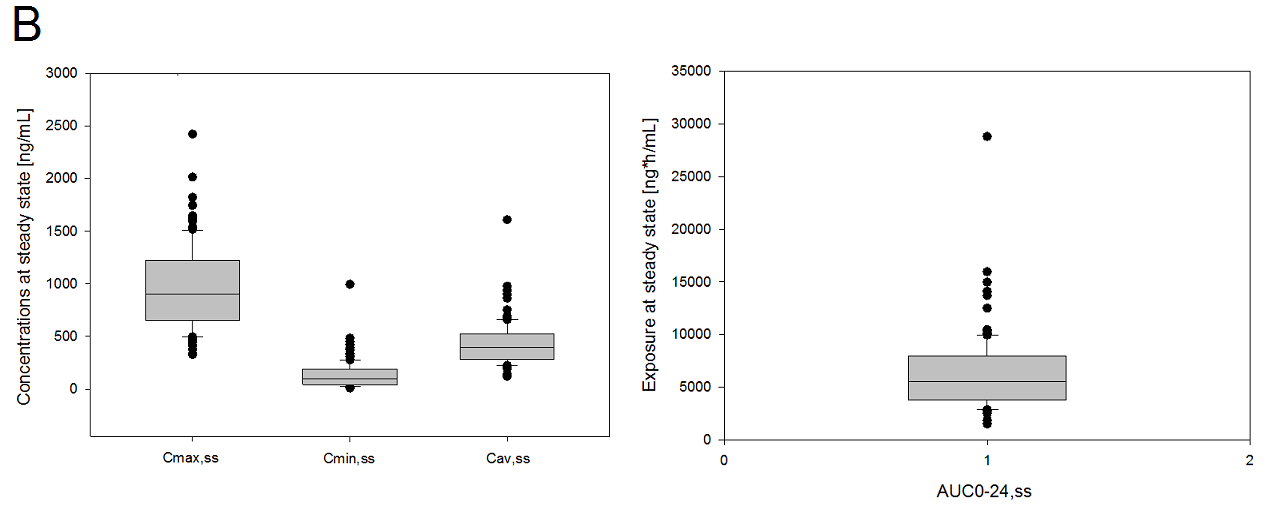
**Fig. S5. Pharmacokinetic profile**. (A) Observed plasma galunisertib concentrations following single dose and 150 mg BID multiple-dose administration for 14 days. (B) Box plots of predicted individual maximum, minimum, and average concentrations at steady state, time to maximum concentration at steady state and exposure (AUC_0-24_) at steady state . AUC_0-24,_ss=area under the curve exposure from 0 to 24 hours at steady state. BID=twice per day. C_av,ss_= average concentration at steady state; C_max,ss_=maximum concentration at steady state; C_min,ss_=minimum concentration at steady-state; Hr=hour; MD=multiple dose; SD=single dose


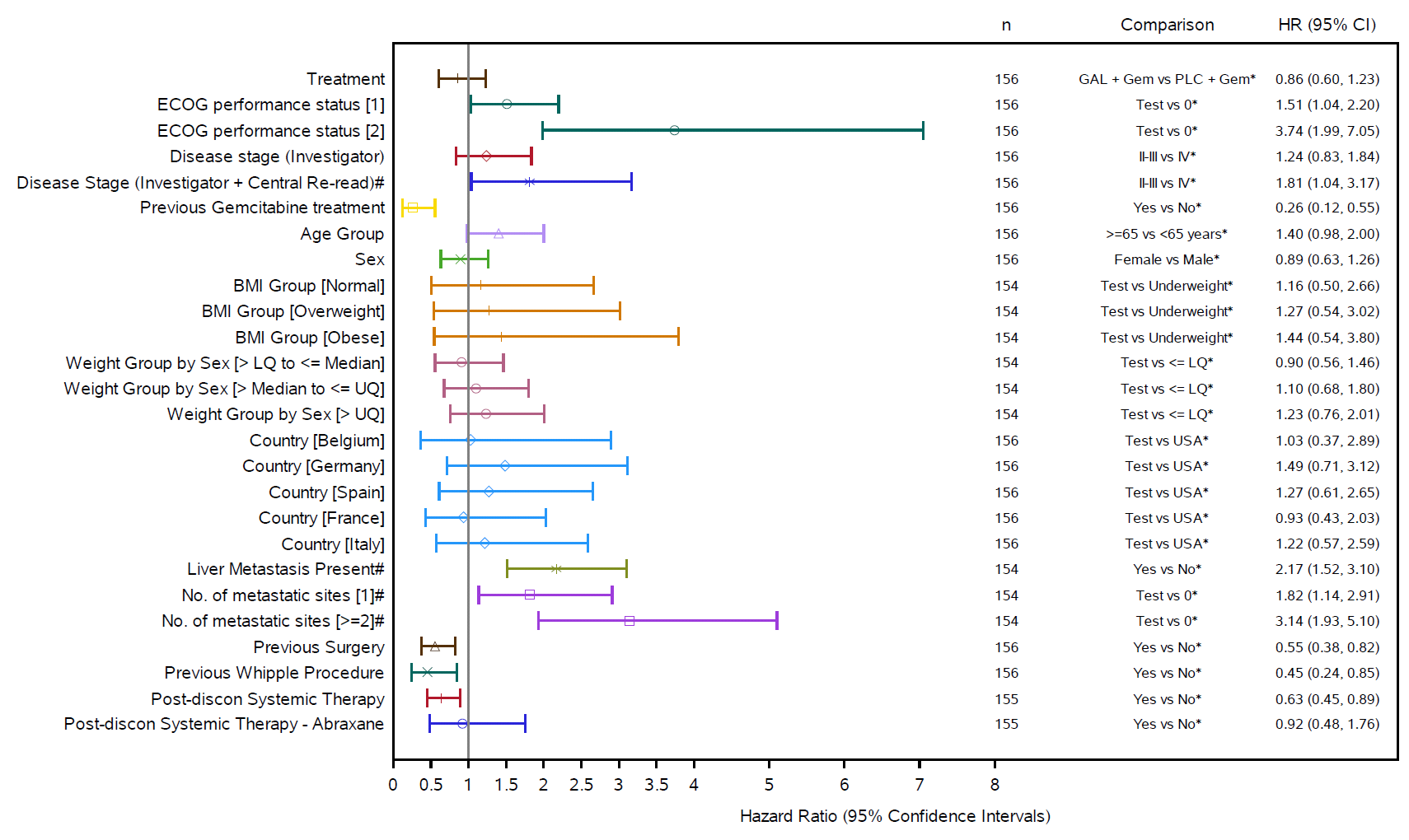


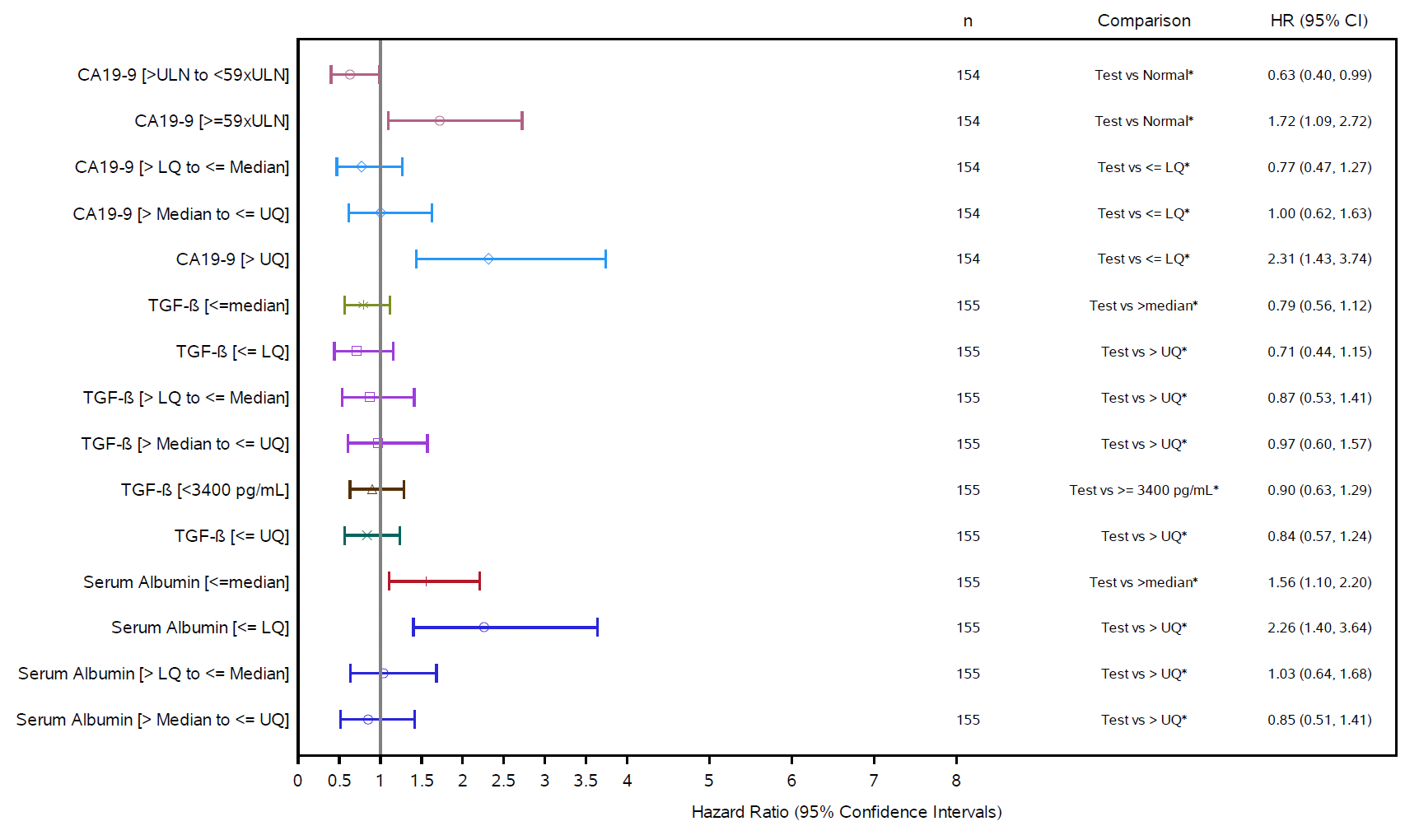


**Fig. S6. Potential prognostic factor effects on overall survival.** BMI=body mass index; CA19-9=carbohydrate antigen 19-9; CI=confidence interval; discon=discontinuation; ECOG=Eastern Cooperative Oncology Group; GAL= galunisertib; GEM=gemcitabine; HR=hazard ratio; LQ=lower quartile; No.=number; PLC=placebo; TGF-β=Transforming growth factor-beta; ULN=upper limit of normal; UQ=upper quartile; USA=United States of America


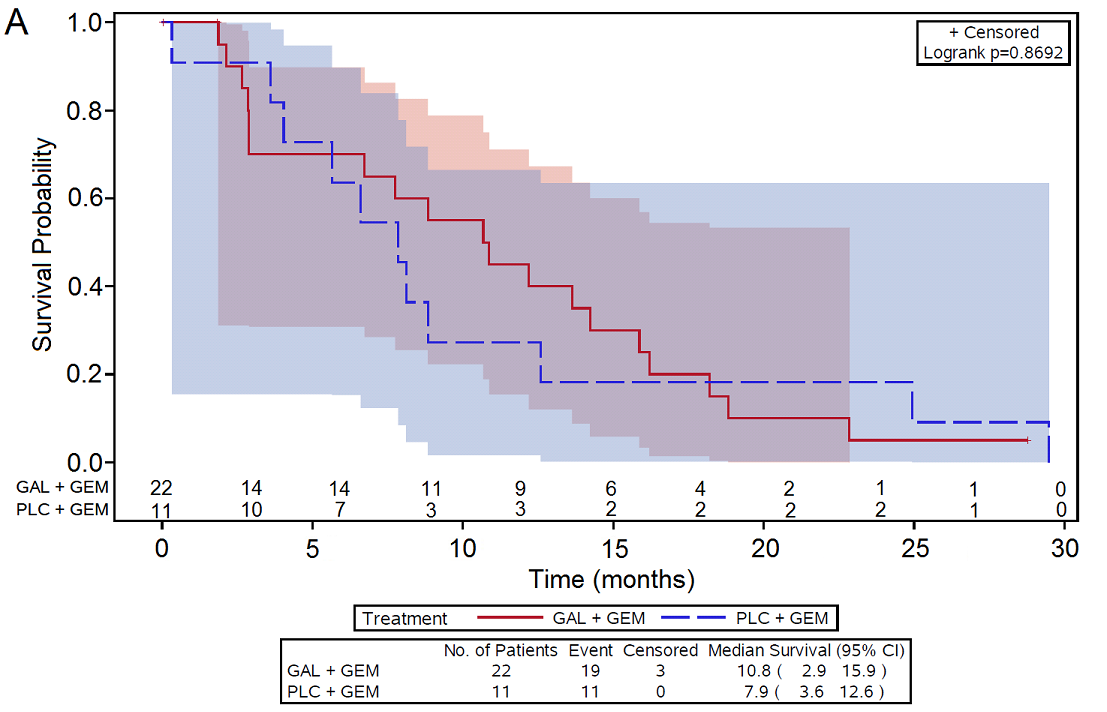


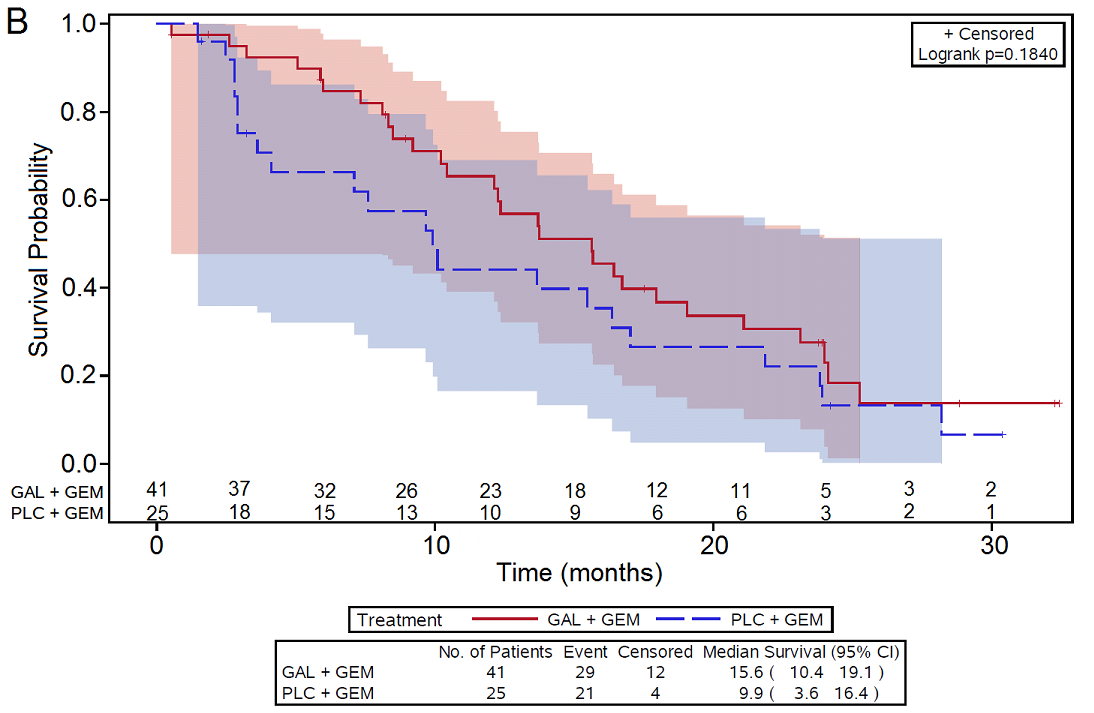


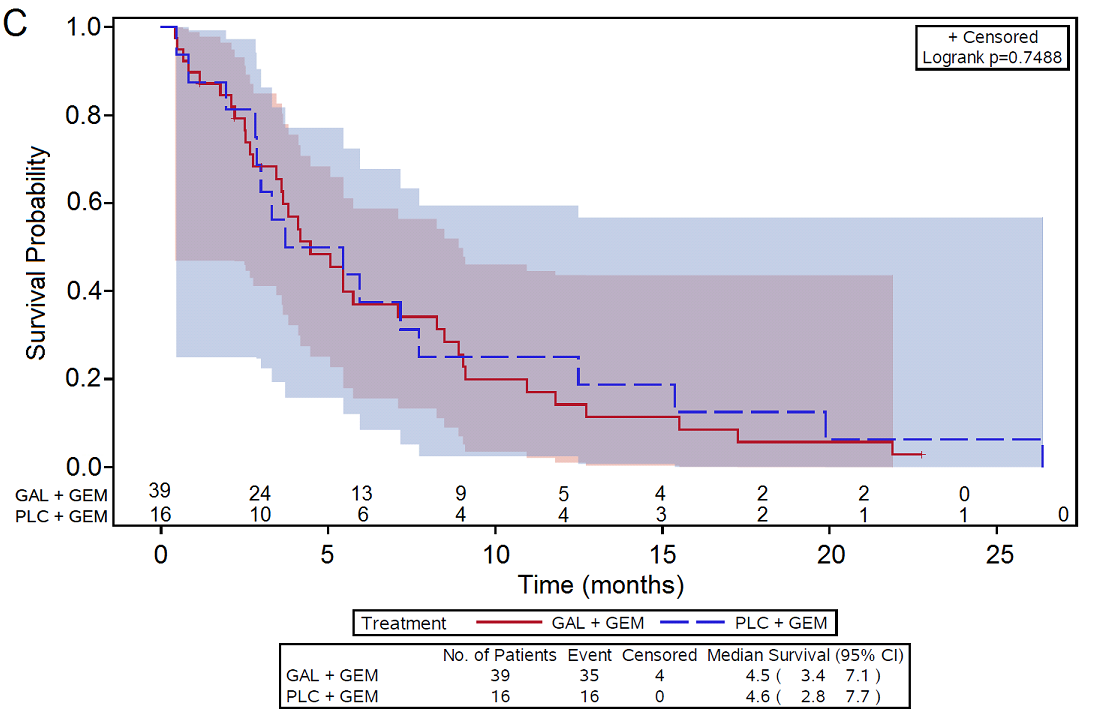


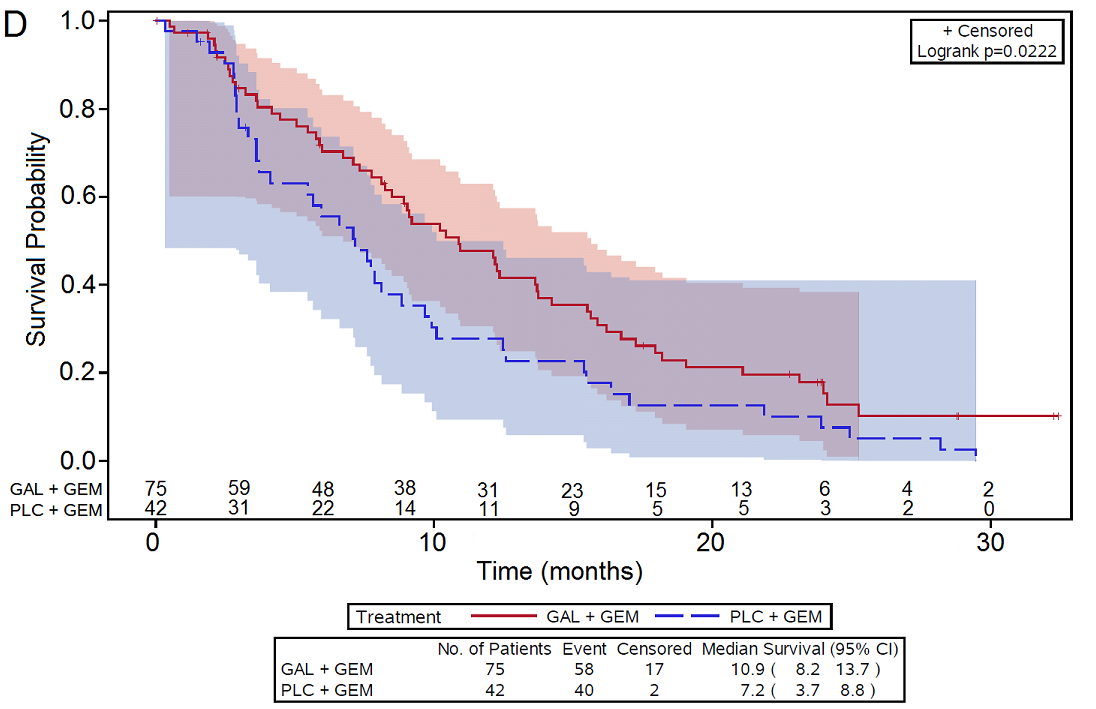


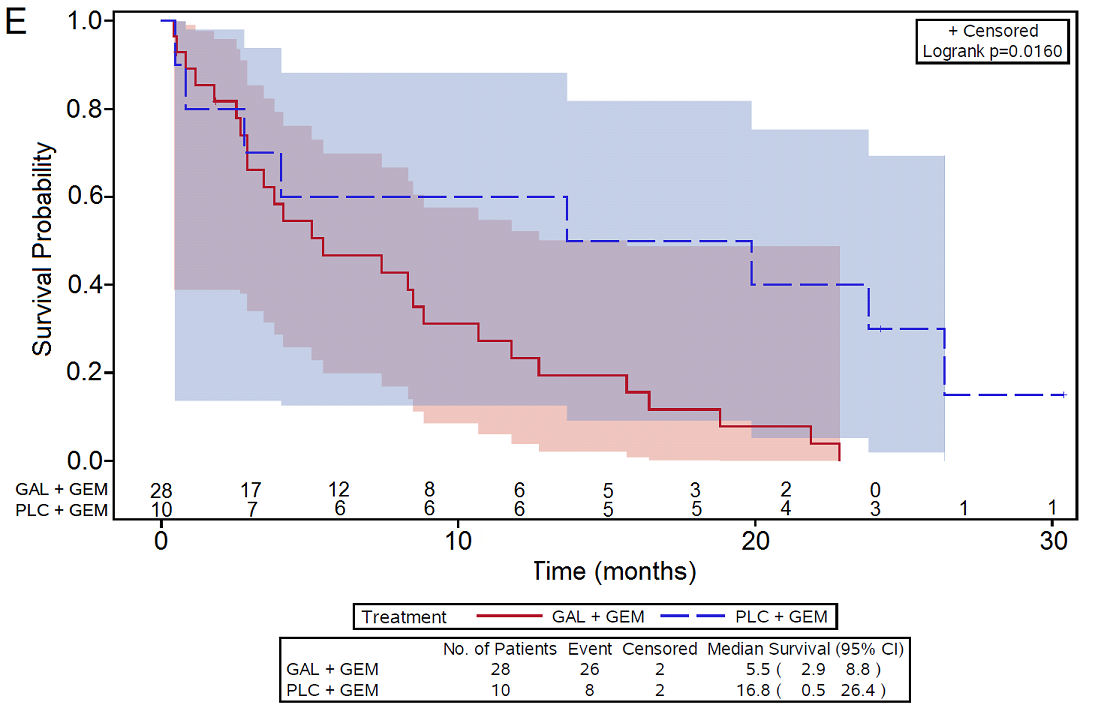


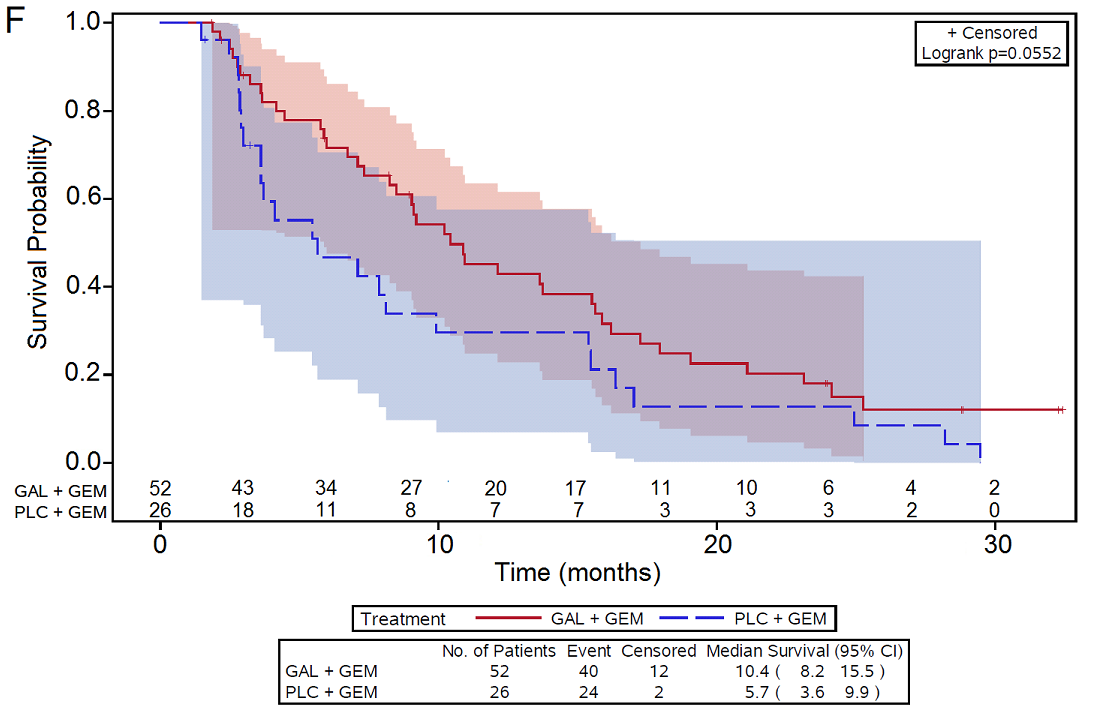


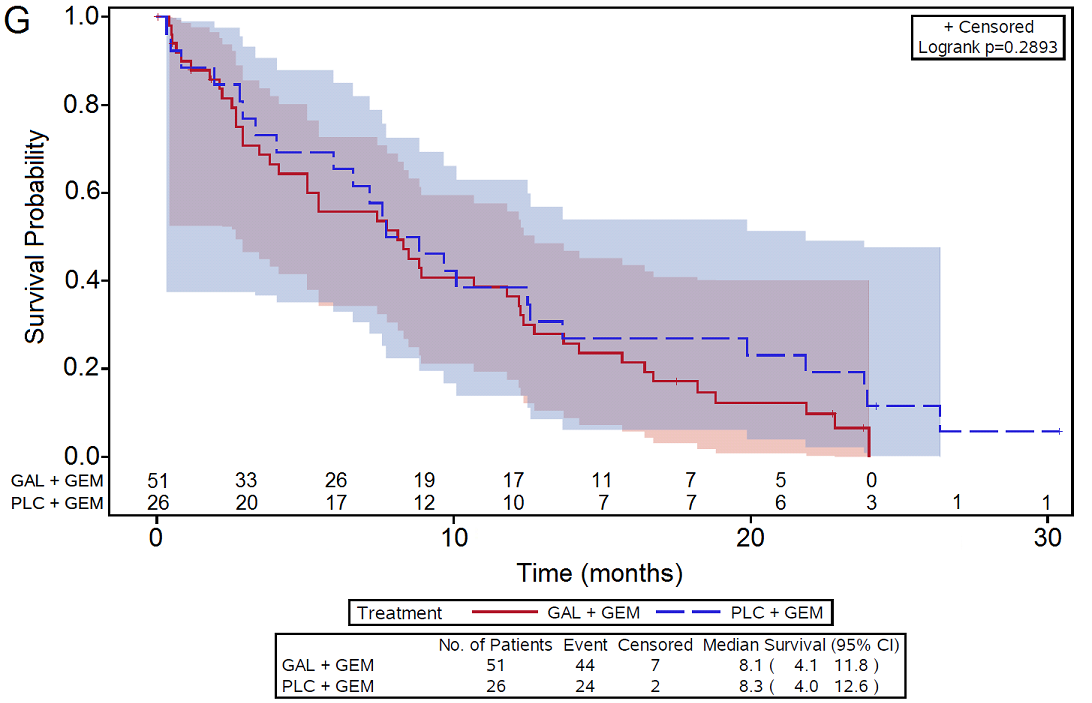


**Fig. S7. Kaplan-Meier estimates of survival by biomarkers, with Hall-Wellner confidence bands and numbers at risk.** Overall survival for patients with (A) CA19-9 within normal limits at baseline, (B) CA19-9 >ULN and <59 x ULN at baseline, (C) CA19-9 ≥59 x ULN at baseline, (D) TGF-β1 ≤UQ, (E) TGF-β1 >UQ, (F) TGF-β1 ≤median, and (G) TGF-β1 >median. CA19-9=carbohydrate antigen 19-9; CI=confidence interval; GAL= galunisertib; GEM=gemcitabine; No.=number; PLC=placebo; TGF-β=Transforming growth factor-beta; ULN=upper limit of normal; UQ=upper quartile


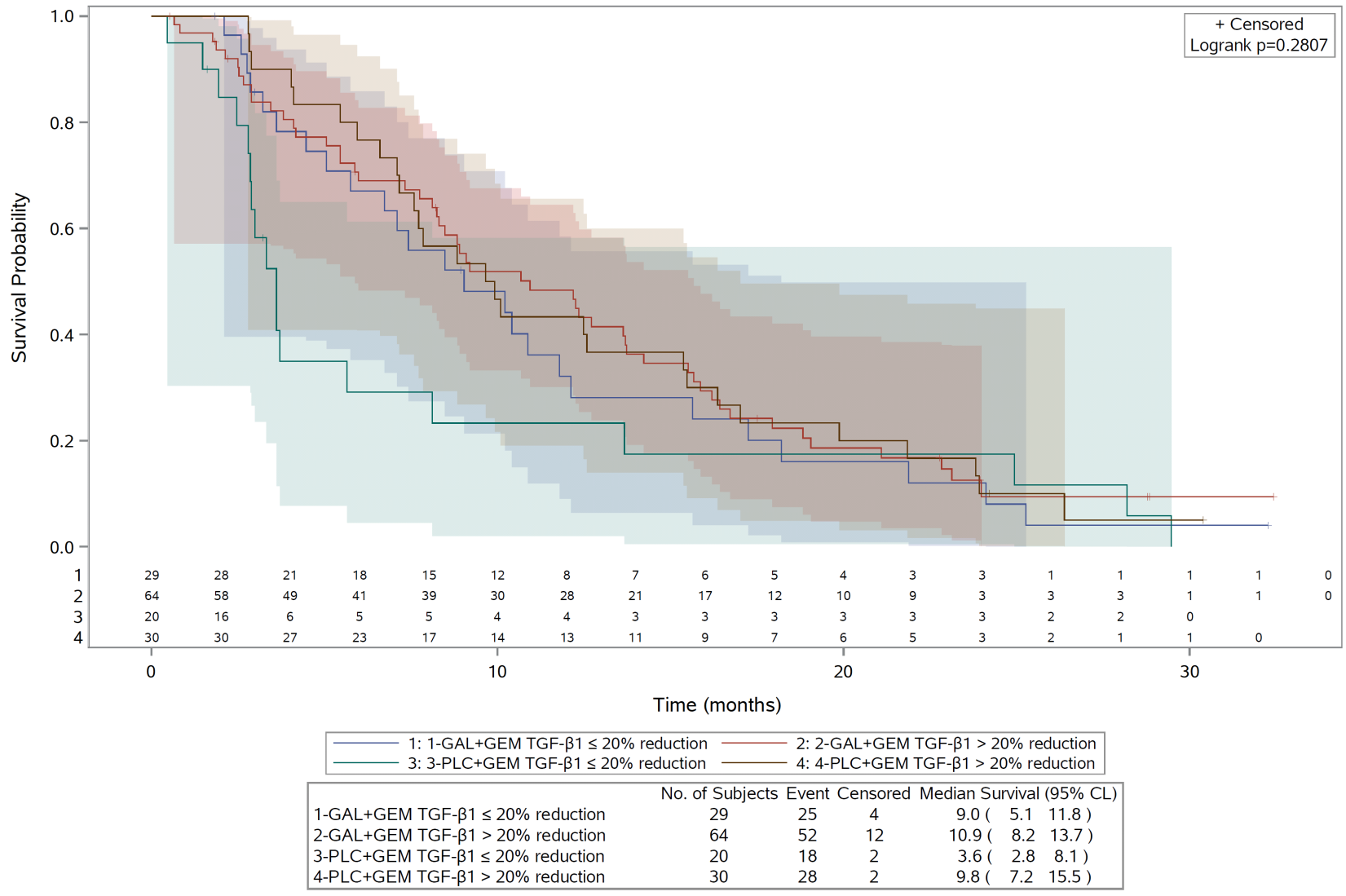


**Fig. S8. Kaplan-Meier estimates of survival by biomarker, with 95% Hall-Wellner confidence bands and numbers at risk.** Overall survival by reduction of TGF-β1. CL=confidence limits; GAL=galunisertib; GEM=gemcitabine; No.=number; PLC=placebo; TGF-β1=transforming growth factor-beta


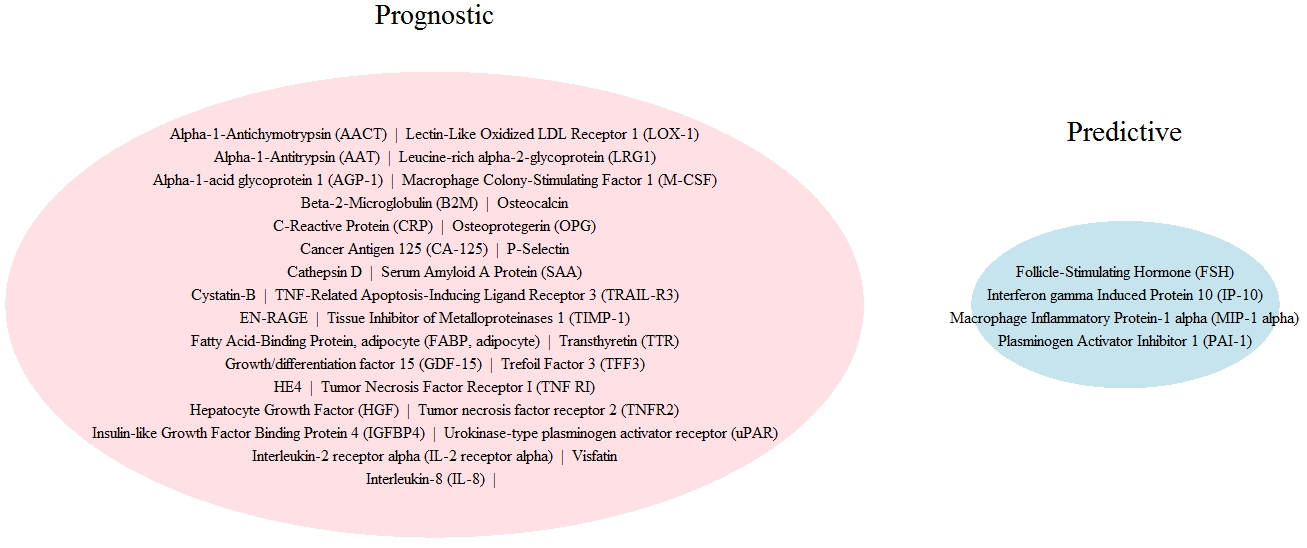


**Fig. S9. Prognostic and predictive markers.** Prognostic (p<0.001) and predictive (p<0.01) potential of proteins based on median cut.

**
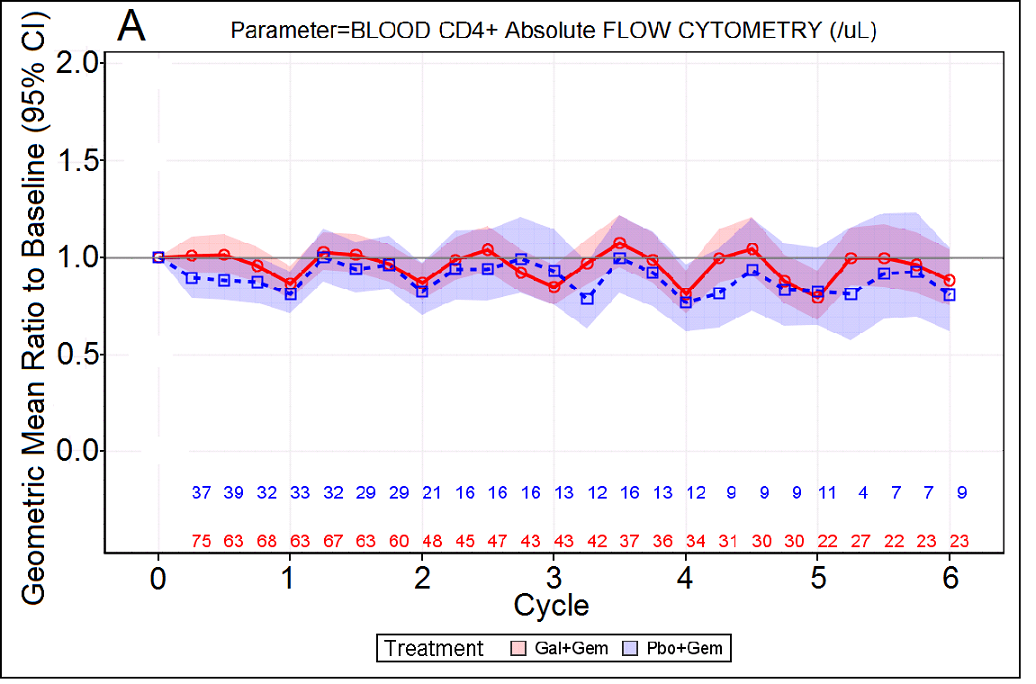
**

**
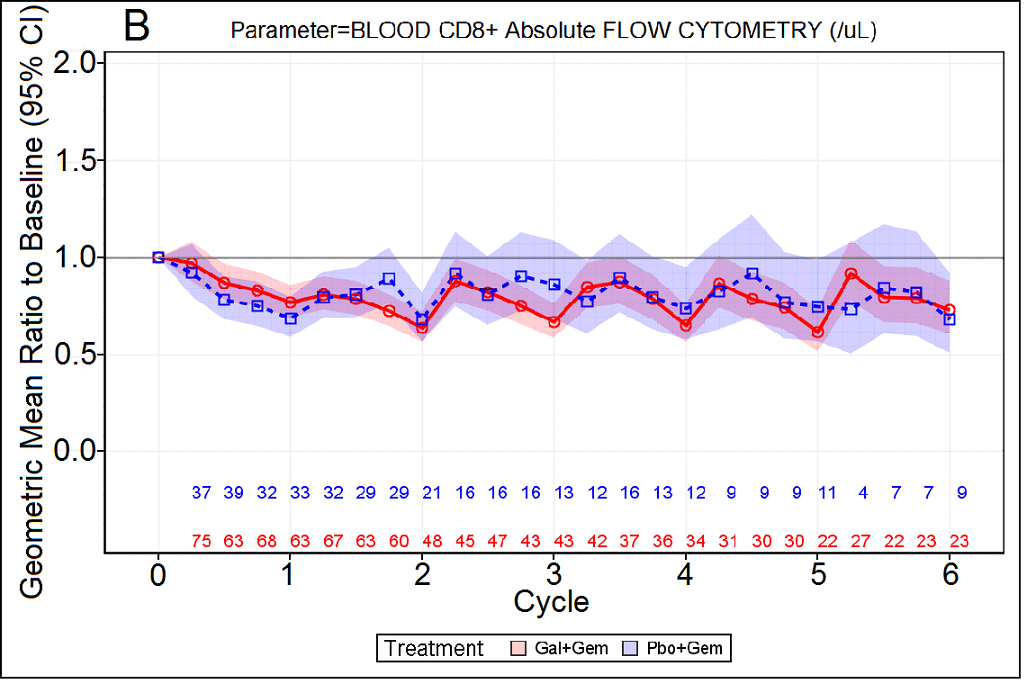
**

**
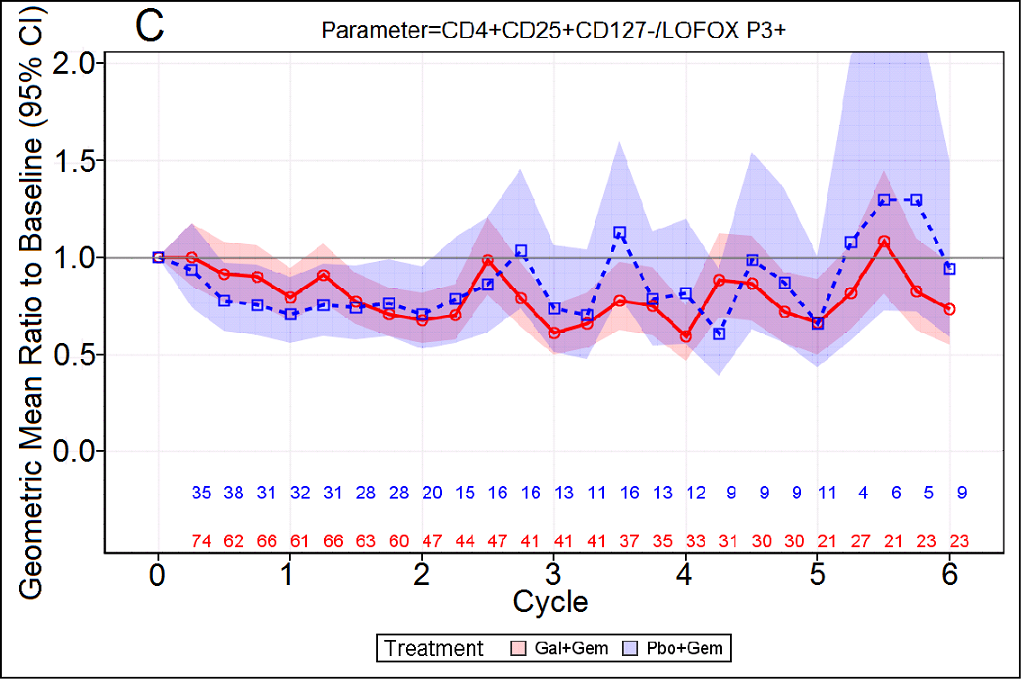
**

**
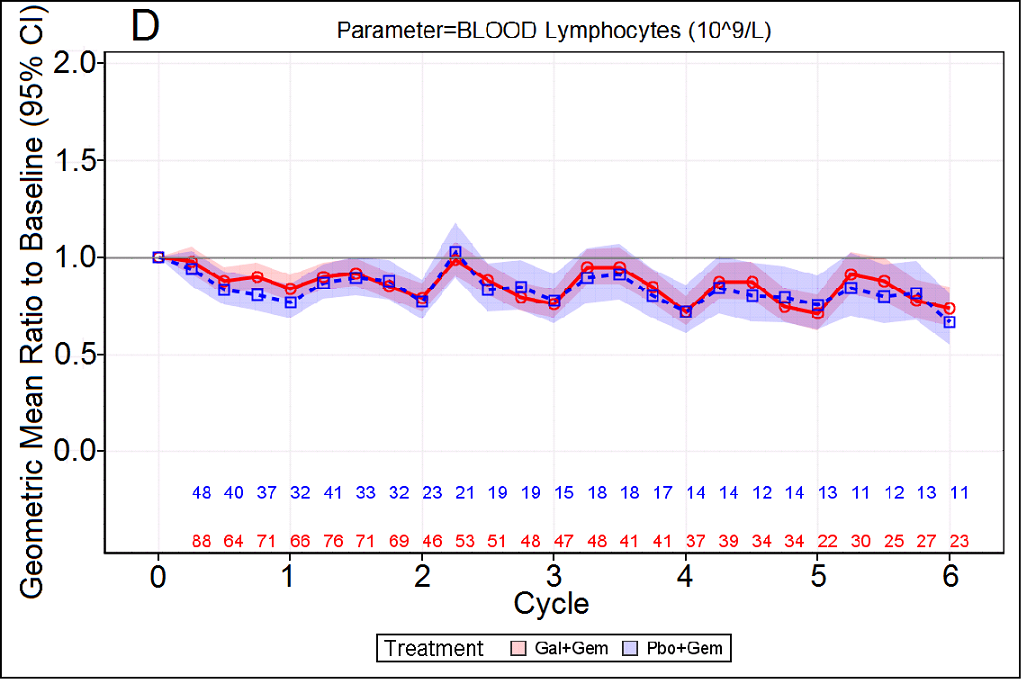
**

**Fig. S10. Immunological laboratory results with 95% confidence bands.** (A) T cell subset CD4. (B) T cell subset CD8. (C) T regulatory cells. (D) Total lymphocyte count. CI=confidence interval; GAL=galunisertib; GEM=gemcitabine; Pbo=placebo
