# Supplementary material for: Galunisertib plus gemcitabine vs. gemcitabine for first-line treatment of patients with unresectable pancreatic cancer
Source: Br J Cancer. 2018 Oct 15;119(10):1208–14. doi: 10.1038/s41416-018-0246-z (PMC6251034; doi:10.1038/s41416-018-0246-z)
Supplement: Supplementary file 3 — Supplementary Tables [file 41416_2018_246_MOESM3_ESM.docx]

**Table S1.** **Comparison of overall survival and prognostic factors impacting Bayesian analysis from current and historical studies of gemcitabine therapy in pancreatic cancer**

|  | Current Study | Historical Study 1^a^ | Historical Study 2^b^ |
| --- | --- | --- | --- |
| Number of patients randomized to gemcitabine alone | 52 | 282 | 67 |
| Number of events (deaths) | 48 | 234 | 62 |
| Median OS (95% CI) (Kaplan-Meier estimate) | 7.6  (4.0–9.9) | 6.3  (5.4–6.9) | 8.3  (6.5–10.8) |
| Disease stage, *N* (%) | | | |
| II-III  IV | 16 (31%)  36 (69%) | 25 (9%)  257 (91%) | 7 (10%)  60 (90%) |
| ECOG status | | | |
| 0  1  2 | 19 (37%)  29 (56%)  4 (8%) | 86 (30%)  161 (57%)  22 (8%) | 21 (31%)  39 (58%)  7 (10%) |
| Previous gemcitabine therapy | 4 (8%) | 0 | 0 |

^a^ As reported by Oettle and colleagues^2^ and patient-level data provided by Lilly.

^b^ As reported by Saif and colleagues.^3^

CI=confidence interval; ECOG=Eastern Cooperative Oncology Group; OS=overall survival

**Table S2**. **Patient baseline characteristics**

|  | **Phase 1b** | | | **Phase 2** | | |
| --- | --- | --- | --- | --- | --- | --- |
|  | **Galunisertib 80 mg/day + gemcitabine**  **(*N*=5)** | **Galunisertib 160 mg/day + gemcitabine**  **(*N*=4)** | **Galunisertib 300 mg/day + gemcitabine**  **(*N*=5)** | **Placebo group**  **(*N*=52)** | **Galunisertib group**  **(*N*=103)^d^** | **Total**  **(*N*=155)** |
| Tumor types |  |  |  |  |  |  |
| Adenocarcinoma, not otherwise specified | 2 (40%) | 0 | 0 |  |  |  |
| CRC (incl rectal Ca) | 2 (40%) | 0 | 2 (40%) |  |  |  |
| NSCLC/lung | 1 (20%) | 1 (25%) | 0 |  |  |  |
| Pancreatic cancer | 0 | 1 (25%) | 3 (60%) | 52 (100%) | 103 (100%) | 155 (100%) |
| Oesophageal cancer | 0 | 1 (25%) | 0 |  |  |  |
| Adenocarcinoma, well differentiated | 0 | 1 (25%) | 0 |  |  |  |
| Median age, years | 70.0 | 65.0 | 57.0 | 66.0 | 68.0 | 67.0 |
| Range | 41–72 | 52–73 | 43–68 | 45–81 | 49–84 | 45–84 |
| Sex |  |  |  |  |  |  |
| Female | 0 | 3 (75%) | 3 (60%) | 24 (46%) | 46 (45%) | 70 (45%) |
| Male | 5 (100%) | 1 (25%) | 2 (40%) | 28 (54%) | 57 (55%) | 85 (55%) |
| Race^a^ |  |  |  |  |  |  |
| White | 3 (60%) | 4 (100%) | 5 (100%) | 50 (100%) | 95 (100%) | 145 (100%) |
| African American | 2 (40%) | 0 | 0 | 0 | 0 | 0 |
| Missing | 0 | 0 | 0 | 2 | 8 | 10 |
| ECOG PS |  |  |  |  |  |  |
| 0 | 3 (60%) | 2 (50%) | 2 (40%) | 19 (37%) | 34 (33%) | 53 (34%) |
| 1 | 2 (40%) | 1 (25%) | 3 (60%) | 29 (56%) | 58 (56%) | 87 (56%) |
| 2 | 0 | 1 (25%) | 0 | 4 (8%) | 11 (11%) | 15 (10%) |
| Disease stage at study entry^b^ |  |  |  |  |  |  |
| II | 0 | 0 | 0 | 1 (2%) | 4 (4%) | 5 (3%) |
| III | 0 | 0 | 0 | 6 (12%) | 9 (9%) | 15 (10%) |
| IV | 5 (100%) | 4 (100%) | 5 (100%) | 45 (87%) | 91 (88%) | 136 (87%) |
| Number of prior treatments |  |  |  |  |  |  |
| 1 regimen | 0 | 1 (25%) | 0 | 1 (2%) | 3 (3%) | 4 (3%) |
| 2 regimens | 1 (20%) | 3 (75%) | 1 (20%) | 3 (6%) | 5 (5%) | 8 (5%) |
| ≥3 regimens | 4 (80%) | 0 | 4 (80%) | 1 (2%) | 2 (2%) | 3 (2%) |
| Number of patients who had at least 1 prior surgery | 4 (80%) | 1 (25%) | 2 (40%) | 16 (31%) | 35 (34%) | 51 (33%) |
| Potential prognostic factors^c^ |  |  |  | (n=52) | (n=104) | (n=156) |
| Liver metastases present |  |  |  | 21 (41%) | 56 (54%) | 77 (50%) |
| Number of metastatic sites^a^ |  |  |  |  |  |  |
| 0 |  |  |  | 15 (29%) | 24 (23%) | 39 (25%) |
| 1 |  |  |  | 19 (37%) | 46 (45%) | 65 (42%) |
| ≥2 |  |  |  | 17 (33%) | 33 (32%) | 50 (32%) |
| Missing |  |  |  | 1 | 1 | 2 |
| Level of CA19-9 |  |  |  |  |  |  |
| Normal |  |  |  | 11 (21%) | 22 (22%) | 33 (21%) |
| ULN to <59x ULN |  |  |  | 25 (48%) | 41 (40%) | 66 (43%) |
| ≥59x ULN |  |  |  | 16 (31%) | 39 (38%) | 55 (36%) |
| Missing |  |  |  | 0 | 2 | 2 |
| Level of TGF-β1 (pg/mL) |  |  |  |  |  |  |
| Median (range) |  |  |  | 2649  (73–36,233) | 2598  (57–34,344) | 2641  (57–36,233) |

^a^ Phase 2 characteristic percentage calculated using patients with data; excludes those with missing data.

^b^ Based on a central re-read of scans to determine metastasis at baseline. For disease stage, if metastasis was present according to the independent assessor then the patient was classified as disease stage IV at entry, otherwise the investigator recorded disease stage was used.

^c^ Reported for phase 2 study part only.

^d^ Safety population; one patient died after enrolment, prior to receiving any study drug.

CA19-9=carbohydrate antigen 19-9; ca=cancer; CRC=colorectal cancer; ECOG PS=Eastern Cooperative Oncology Group performance score; incl=including; NSCLC=non-small-cell lung cancer; TGF-β1=transforming growth factor-beta 1; ULN=upper limit of normal

**Table S3. Progression-free survival and best response rate assessments**

|  | Phase 1b | | | Phase 2 | | | | | |
| --- | --- | --- | --- | --- | --- | --- | --- | --- | --- |
|  | **Investigator Determined** | | **Central Reader** | **Investigator**  **Determined** | | | **Central**  **Reader** | | |
|  | Galunisertib + Gemcitabine  (All 3 Cohorts)  *N*=14 | | Galunisertib+ Gemcitabine  (All 3 Cohorts)  *N*=14 | Placebo Group  *N*=52 | | Galunisertib Group  *N*=104 | Placebo Group  *N*=52 | Galunisertib Group  *N*=104 | |
| PFS (phase 2 only) ^a^ |  | |  |  | |  |  |  | |
| Median (95% CI) | - | | - | 2.79  (1.81–3.68) | | 3.61  (2.79–5.39) | 2.86  (1.94–3.75) | 4.11  (2.66–5.42) | |
| HR ^b^ (95% CI) p-value |  | |  | 0.80  (0.56–1.15)  0.2325 | | | 0.85  (0.58–1.26)  0.4193 | | |
| Best response rate | | |  |  |  | |  | |  |
| CR | | 0 | 0 | 0 | 0 | | 0 | | 0 |
| ORR (CR+PR) | | 1 (7%) | 1 (7%) | 3 (6%) | 9 (9%) | | 2 (4%) | | 11 (11%) |
| SD | | 7 (50%) | 5 (36%) | 21 (40%) | 53 (51%) | | 25 (48%) | | 50 (48%) |
| (CBR) CR+PR+SD | | 8 (57%) | 6 (43%) | 24 (46%) | 62 (60%) | | 27 (52%) | | 61 (59%) |

^a^ Median PFS estimated from Kaplan-Meier method. Hazard ratio and p-value from Cox proportional hazards model adjusting for baseline ECOG status, disease stage, and previous gemcitabine treatment.

^b^ HR determined using the safety populations (placebo group: n=52, galunisertib group: n=103).

CBR=clinical benefit rate; CI=confidence interval; CR=complete response; ECOG=Eastern Cooperative Oncology Group; HR=hazard ratio; ORR=overall response rate; PFS=progression-free survival; PR=partial response; SD=stable disease

**Table S4. Investigator-determined drug-related CTCAE events occurring in ≥15% of patients and grade 3/4 events occurring in ≥5% of patients**

|  | Phase 1 | | | Phase 2^a^ | | | | | |
| --- | --- | --- | --- | --- | --- | --- | --- | --- | --- |
|  | **Galunsertib + Gemcitabine (*N*=14)** | | | **Placebo Group (*N*=52)** | | | **Galunisertib Group (*N*=103)** | | |
|  | **Grade 1–2** | **Grade 3** | **Grade 4** | **Grade 1–2** | **Grade 3** | **Grade 4** | **Grade 1–2** | **Grade 3** | **Grade 4** |
| Anemia | 3 (21%) | 3 (21%) | 1 (7%) | 18 (35%) | 7 (13%) | 0 | 29 (28%) | 8 (8%) | 0 |
| Neutrophil count decreased | 1 (7%) | 4 (29%) | 2 (14%) | 6 (12%) | 13 (25%) | 1 (2%) | 5 (5%) | 32 (31%) | 3 (3%) |
| Platelet count decreased | 3 (21%) | 2 (14%) | 1 (7%) | 13 (25%) | 5 (10%) | 1 (2%) | 32 (31%) | 8 (8%) | 0 |
| Nausea | 8 (57%) | 0 | 0 | 14 (27%) | 1 (2%) | 0 | 32 (31%) | 3 (3%) | 0 |
| Vomiting | 7 (50%) | 0 | 0 | 10 (19%) | 2 (4%) | 0 | 19 (18%) | 1 (1%) | 0 |
| Diarrhea | 2 (14%) | 1 (7%) | 0 | 5 (10%) | 0 | 0 | 18 (17%) | 0 | 0 |
| Fever | 4 (29%) | 0 | 0 | 5 (10%) | 0 | 0 | 16 (16%) | 1 (1%) | 0 |
| Edema limbs | 3 (21%) | 0 | 0 | 2 (4%) | 1 (2%) | 0 | 7 (7%) | 0 | 0 |
| Fatigue | 6 (43%) | 2 (14%) | 0 | 12 (23%) | 2 (4%) | 0 | 31 (30%) | 6 (6%) | 0 |
| Anorexia | 3 (21%) | 0 | 0 | 5 (10%) | 1 (2%) | 0 | 14 (14%) | 2 (2%) | 0 (0%) |
| Myalgia | 4 (29%) | 0 | 0 | 1 (2%) | 0 | 0 | 4 (4%) | 0 | 0 |

^a^ Events in phase 2 collected from safety population.

CTCAE=Common Terminology Criteria for Adverse Events.
